# Supplementary material for: Papaverinol-N-Oxide: A Microbial Biotransformation Product of Papaverine with Potential Antidiabetic and Antiobesity Activity Unveiled with In Silico Screening
Source: Molecules. 2023 Feb 7;28(4):1583. doi: 10.3390/molecules28041583 (PMC9963078; doi:10.3390/molecules28041583)
Supplement: Supplementary file 1 [file molecules-28-01583-s001.zip › Supporting information new.pdf]

Supporting information

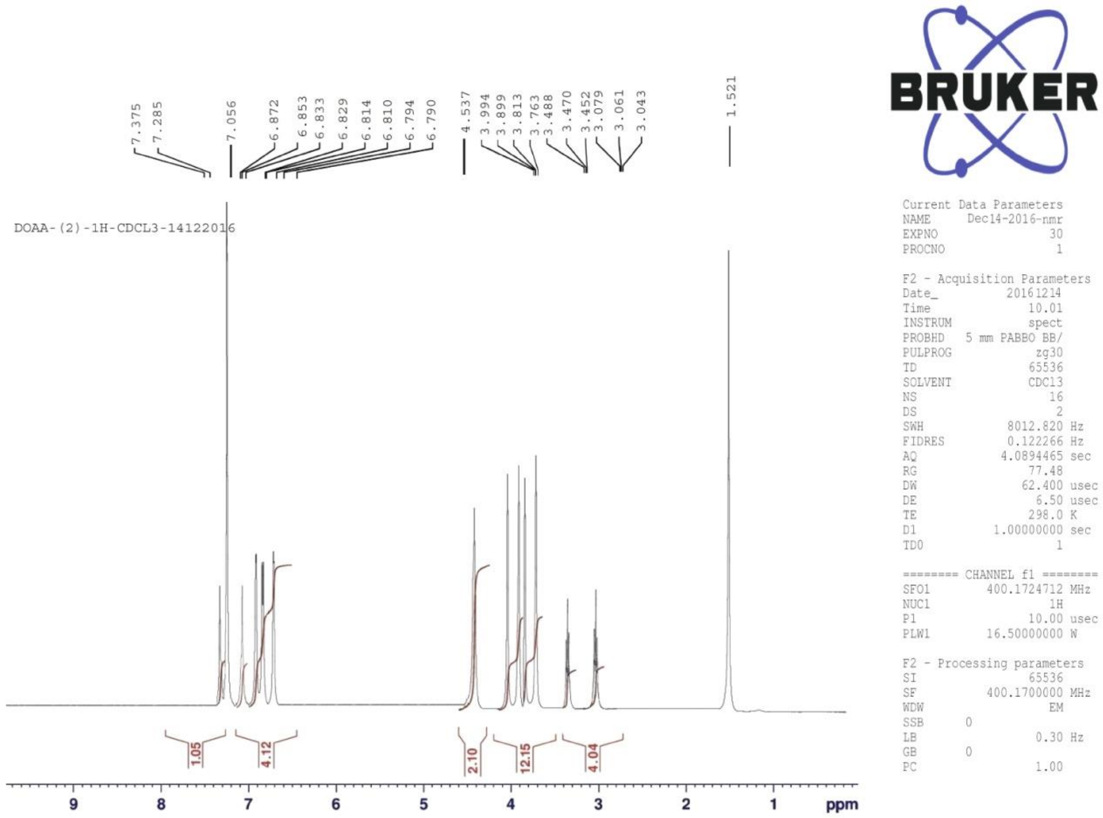

S1: ^{1}H NMR spectrum of metabolite-**1** (400 MHz, $CDCl_3$).

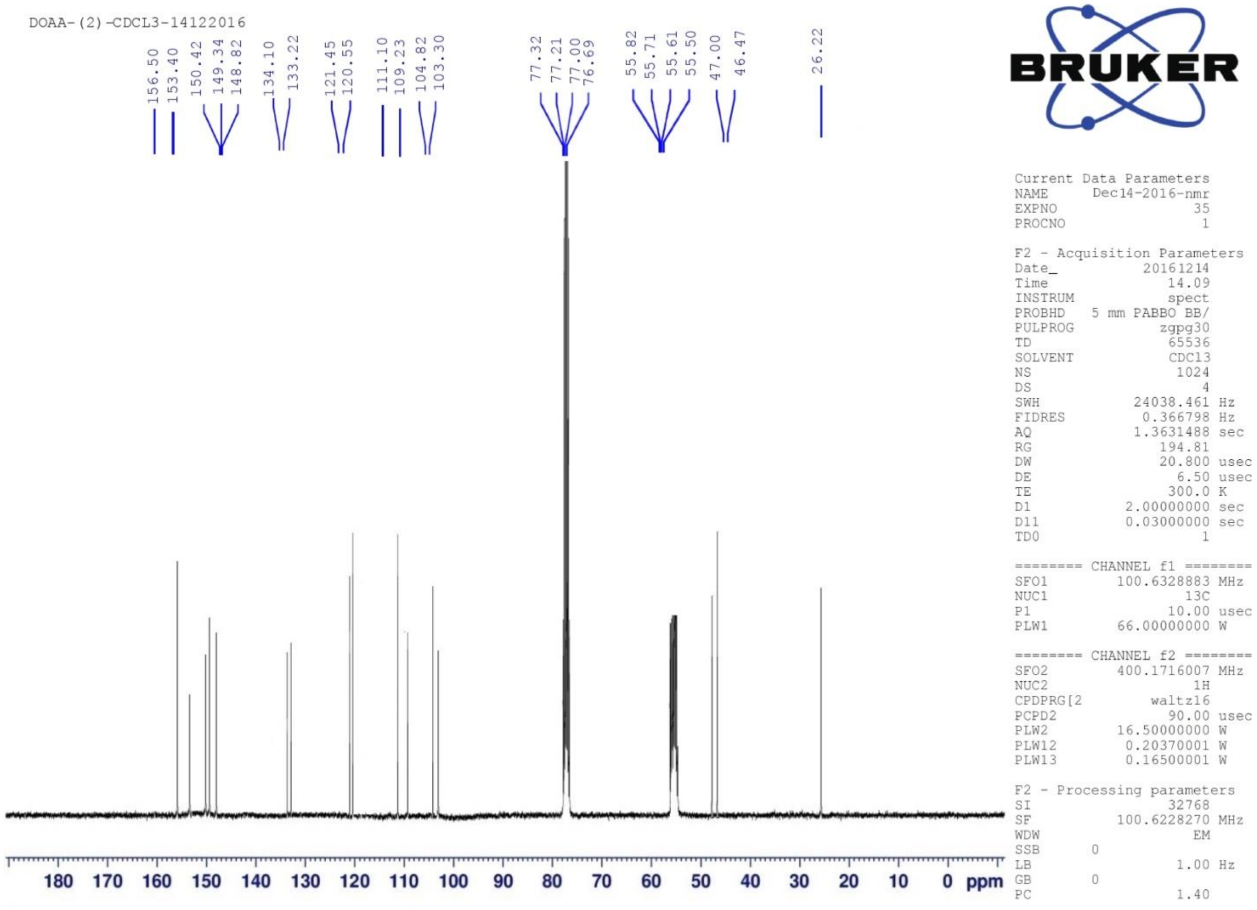

S2: ^{13}C NMR spectrum of metabolite-**1** (100 MHz, $CDCl_3$).

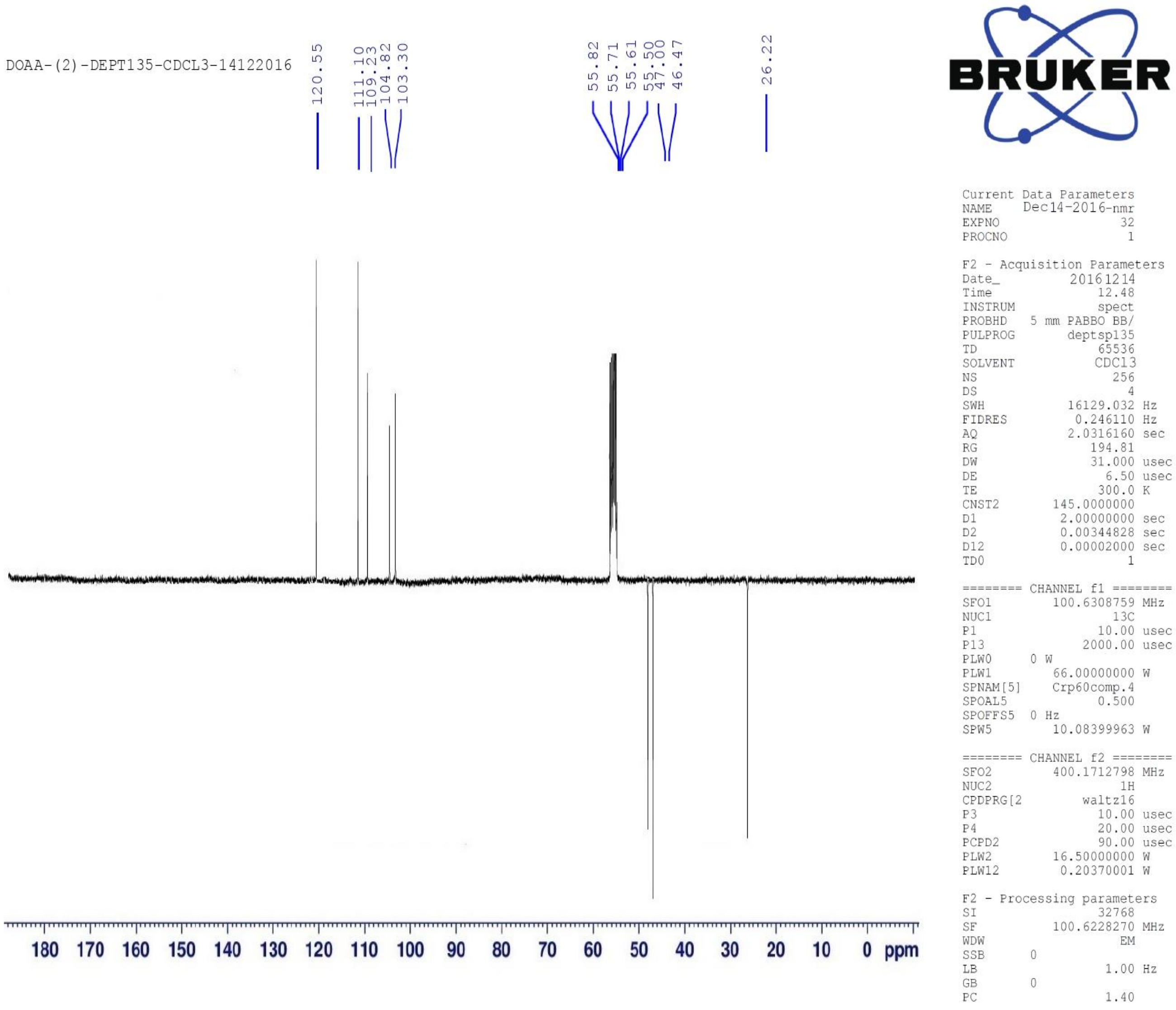

S3: DEPT 135 spectrum of metabolite-**1** (100 MHz, $CDCl_3$).

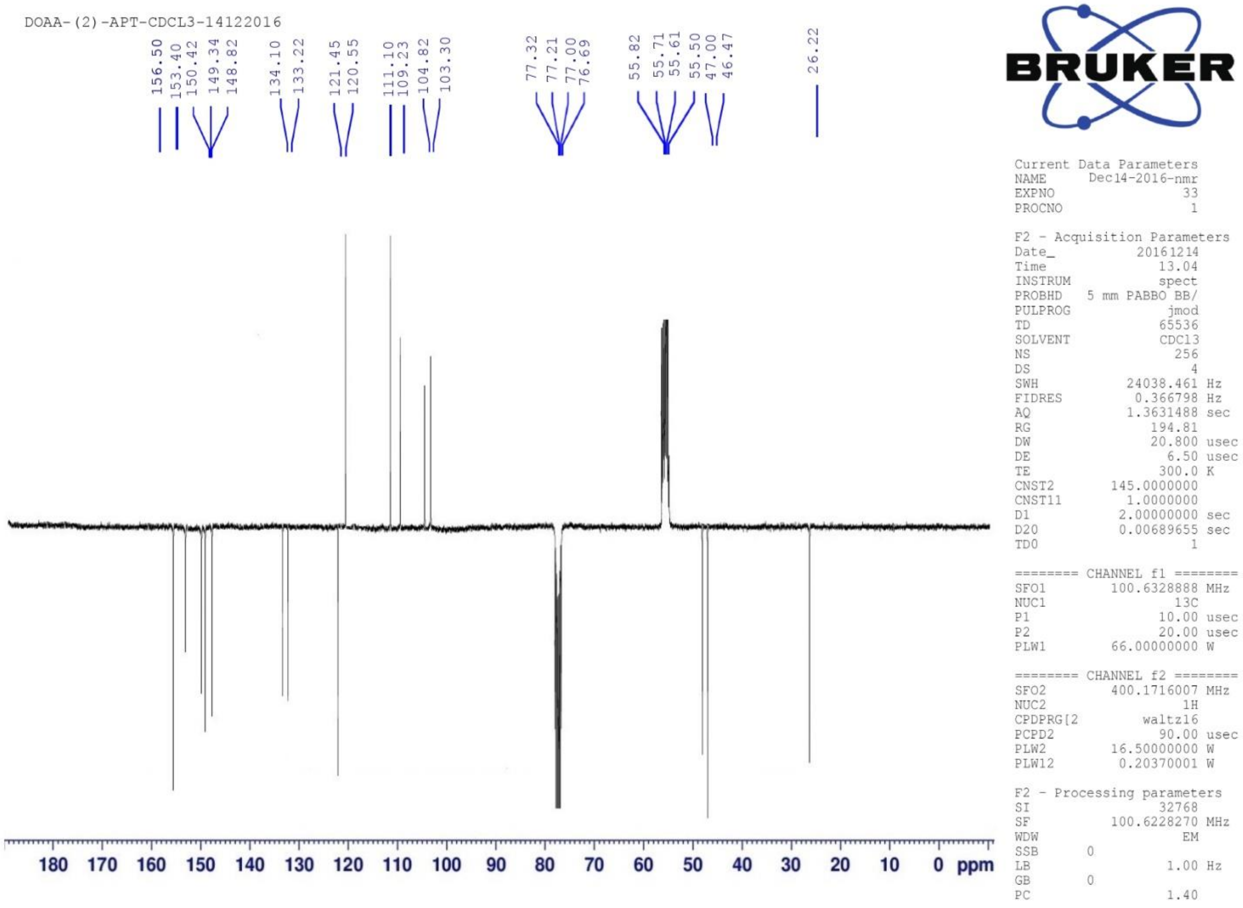

S4: APT spectrum of metabolite-1 (100 MHz, CDCl3).

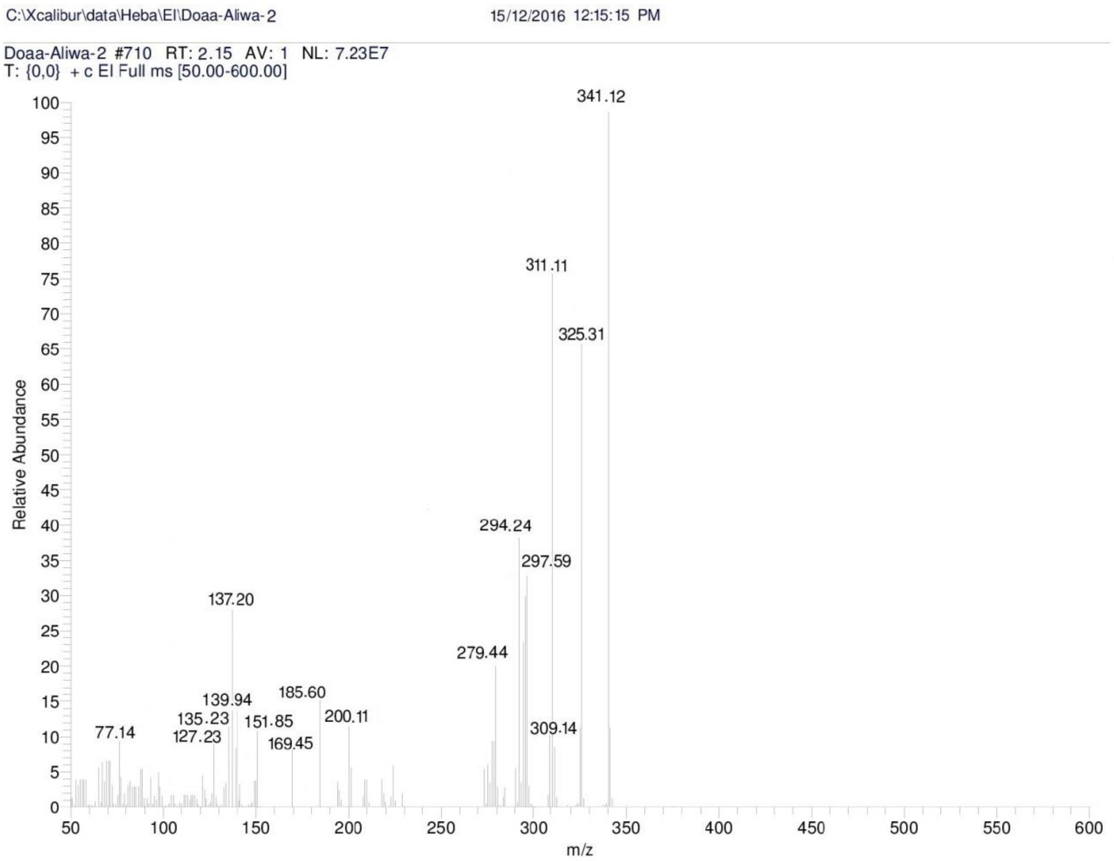

S5: Mass spectrum of **1.**

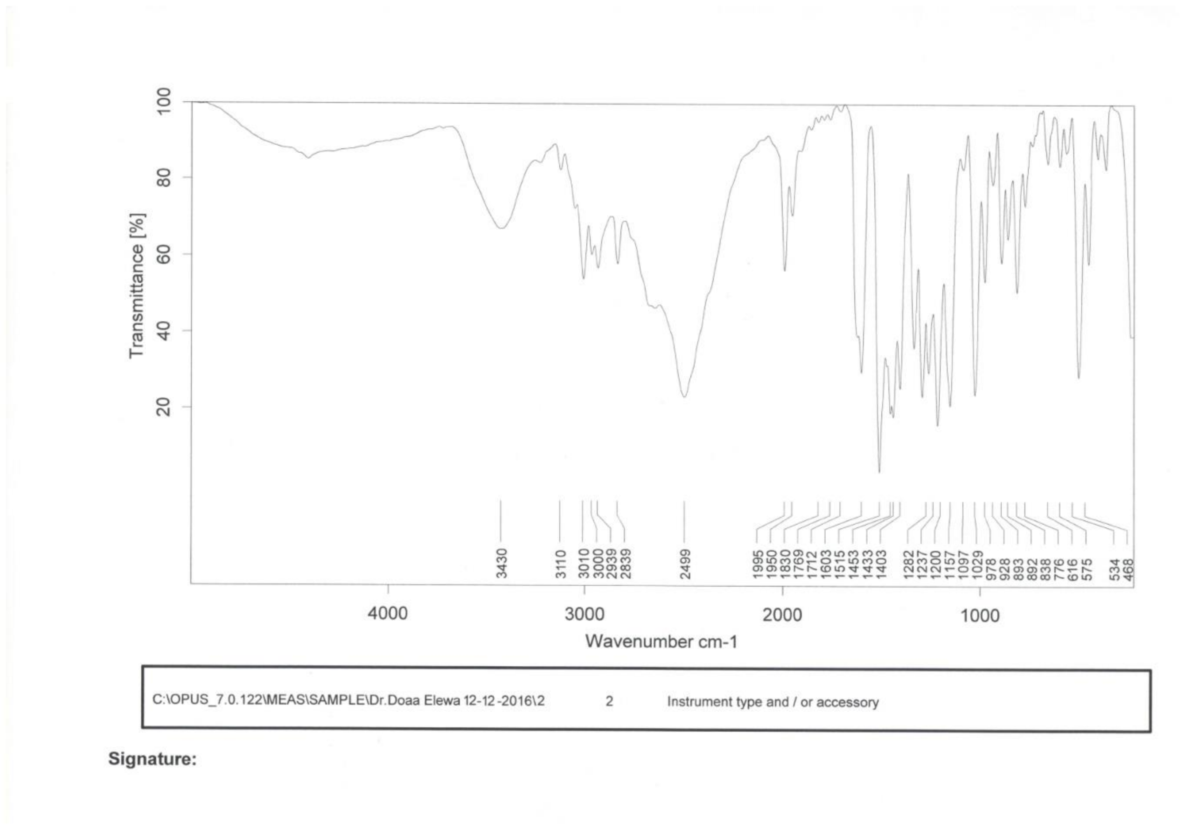

S6: IR spectrum of **1.**

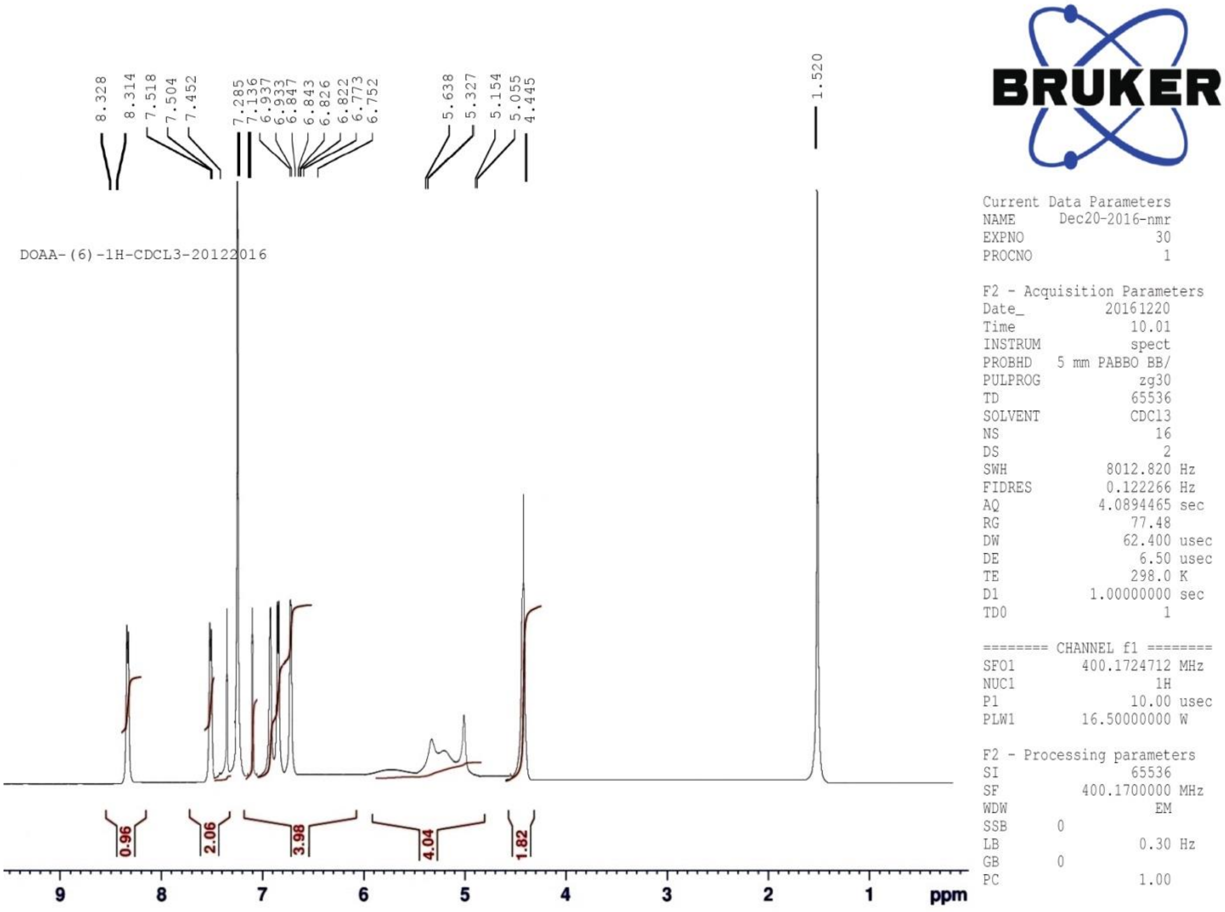

S7: ^{1}H NMR spectrum of metabolite-**2** (400 MHz, $CDCl_3$).

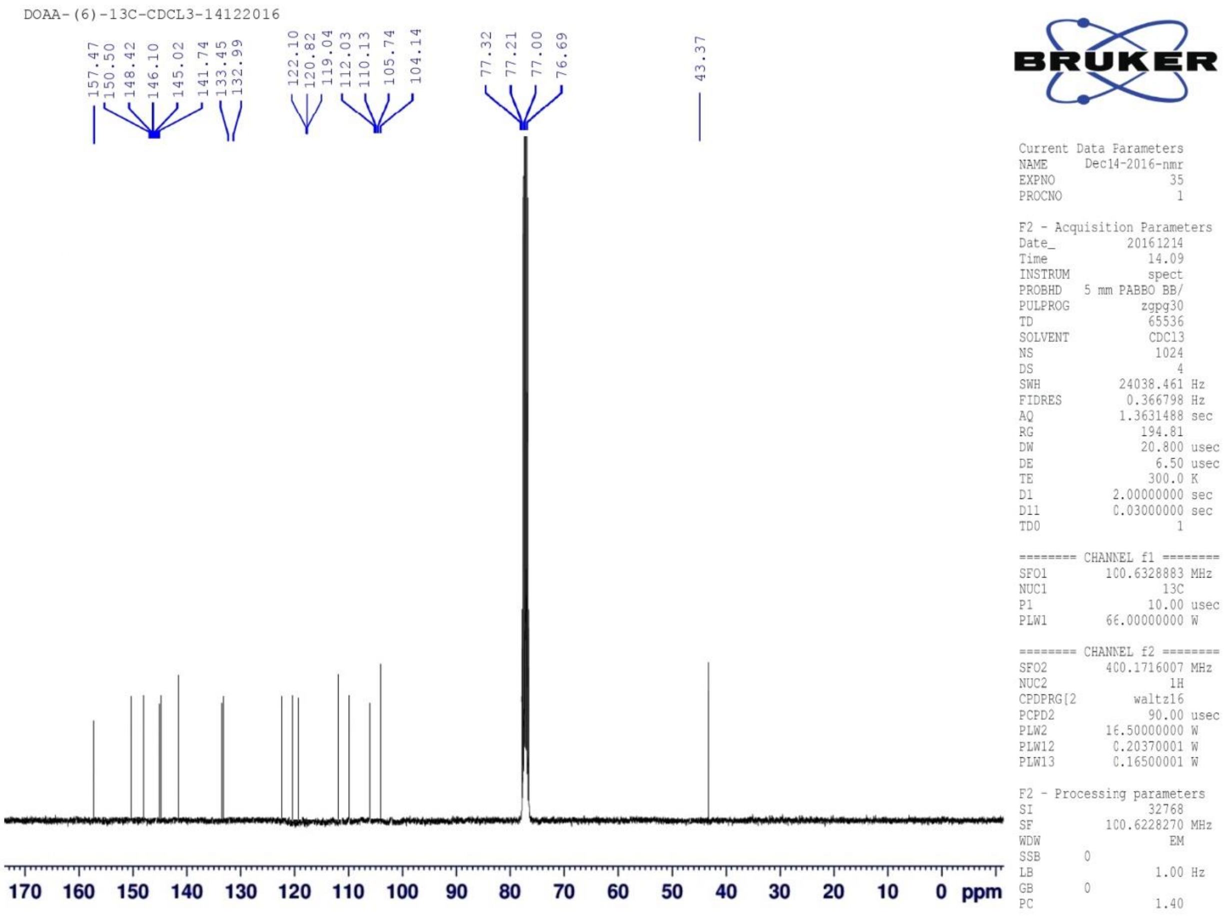

S8: ^{13}C NMR spectrum of metabolite-**2** (100 MHz, $CDCl_3$).

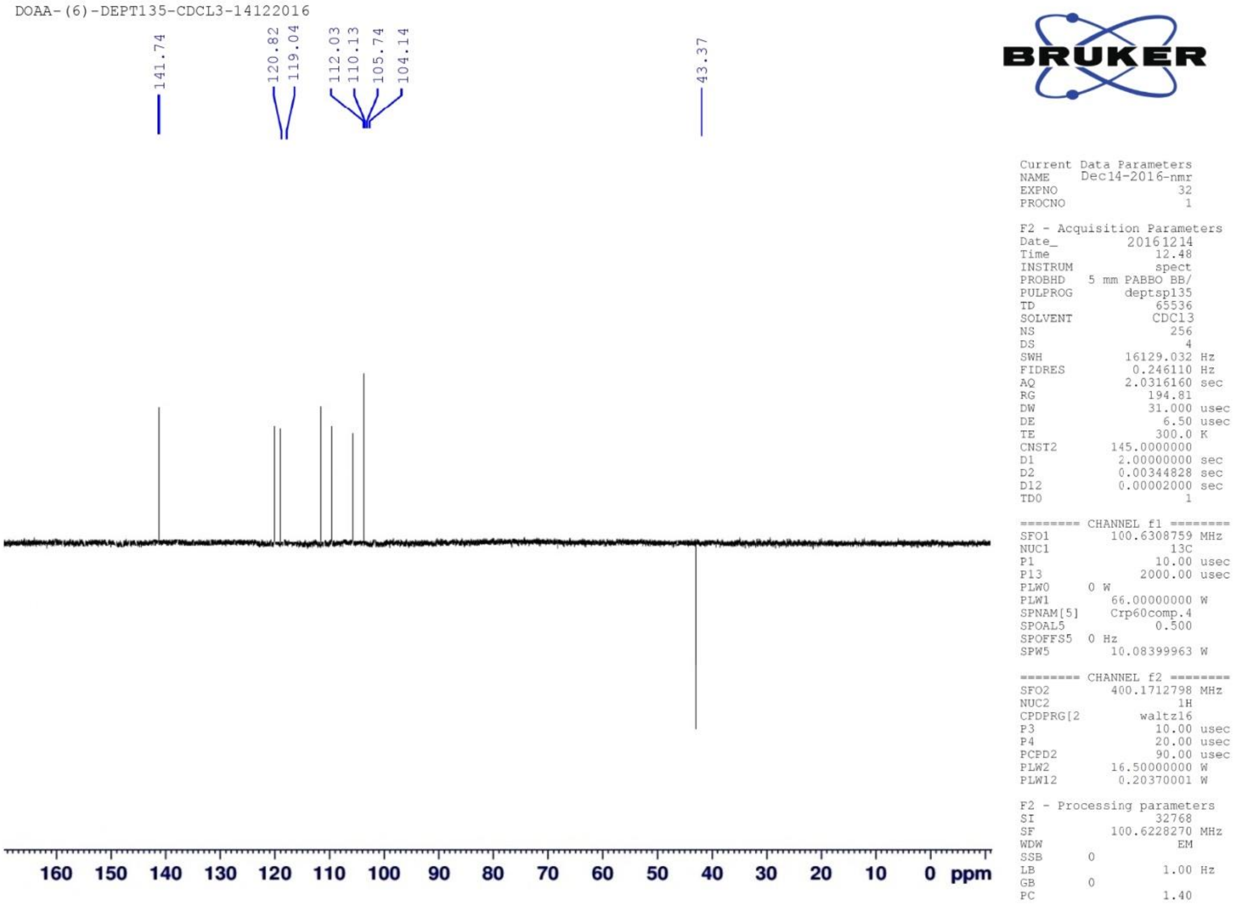

S9: DEPT 135 spectrum of metabolite-**2** (100 MHz, $CDCl_3$).

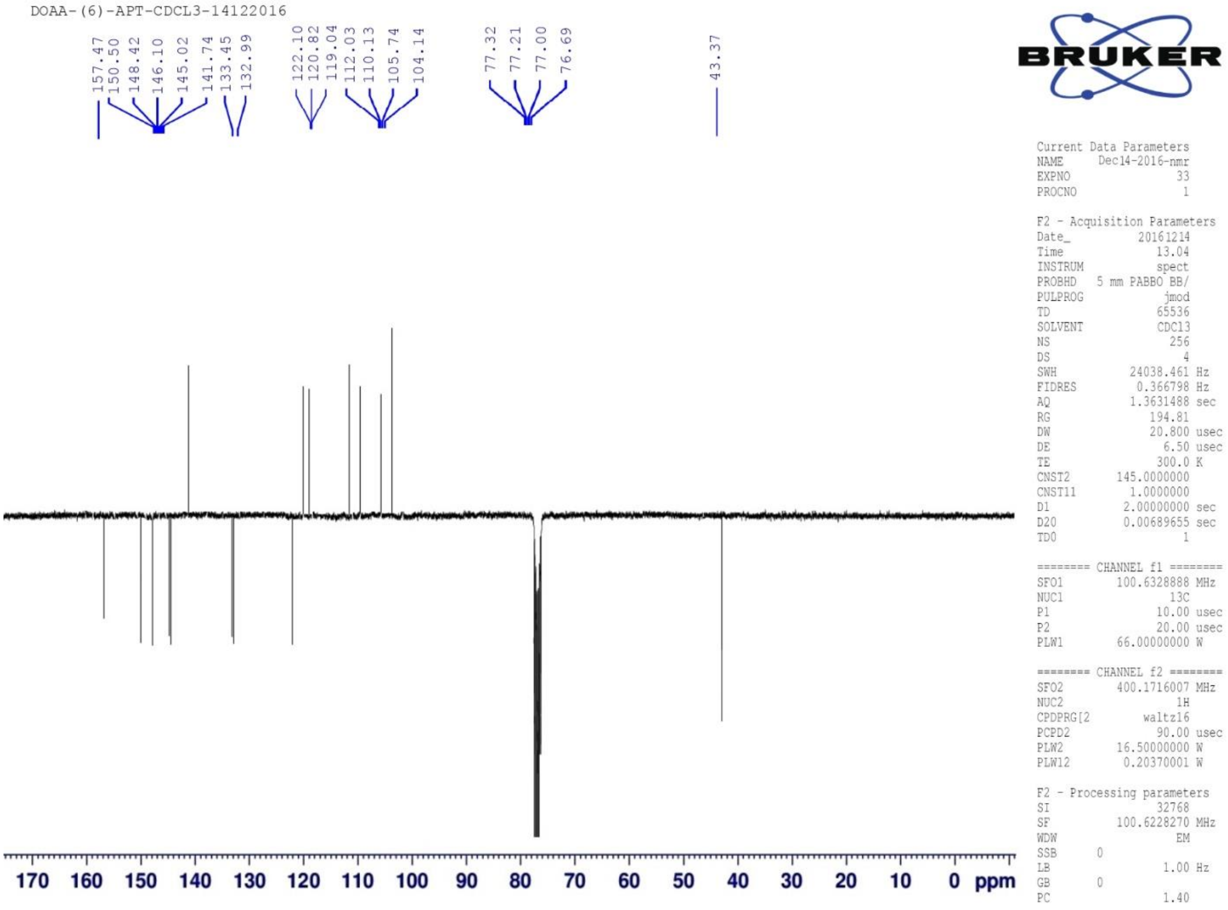

S10: APT spectrum of metabolite-**2** ($CDCl_3$).

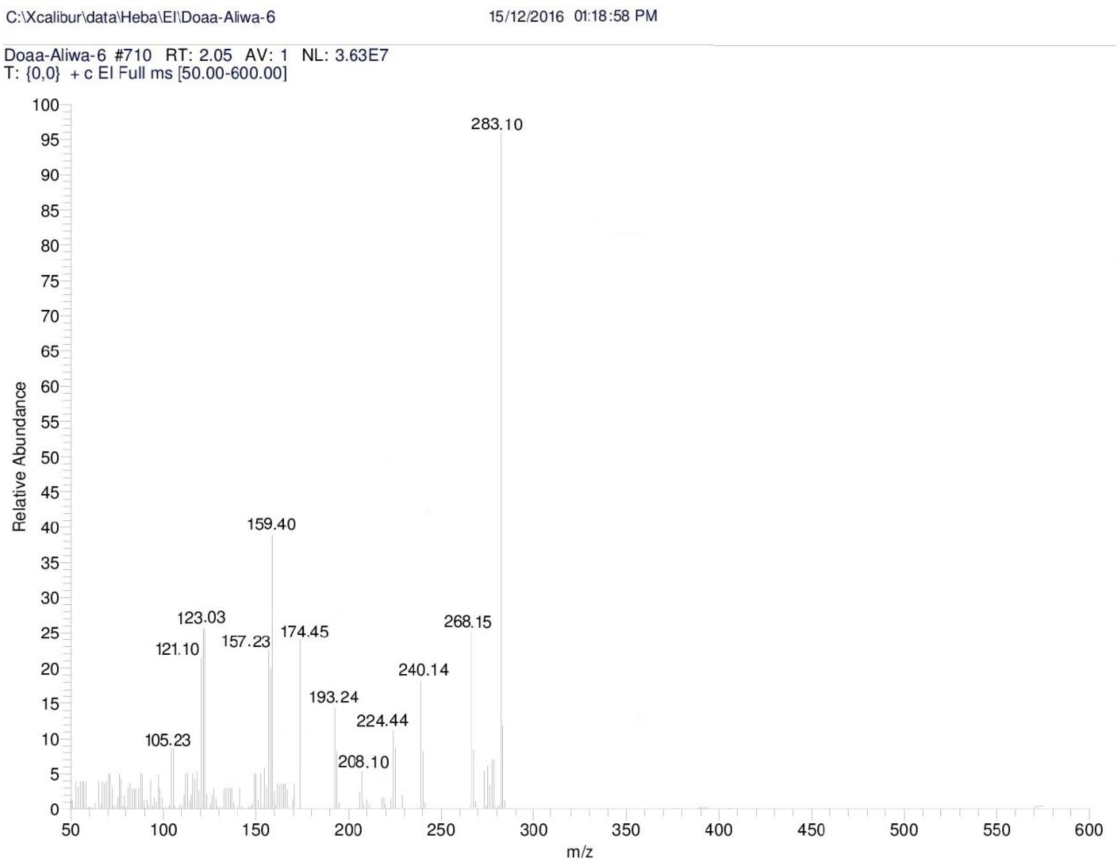

S11: Mass spectrum of the metabolite **-2.**

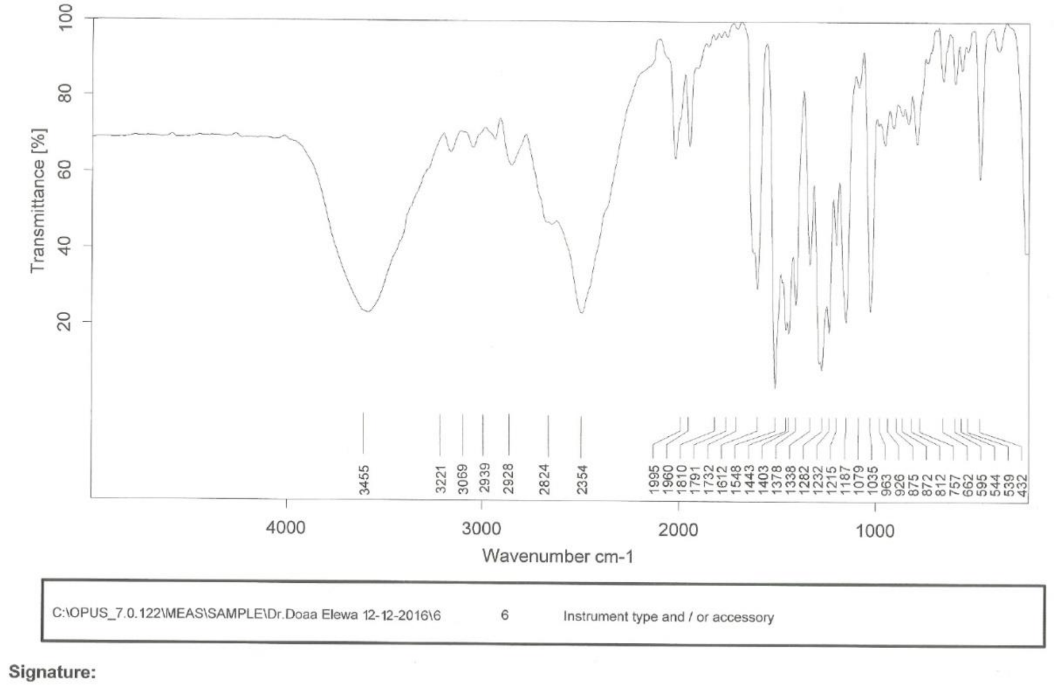

S12: IR spectrum of the metabolite **-2.**

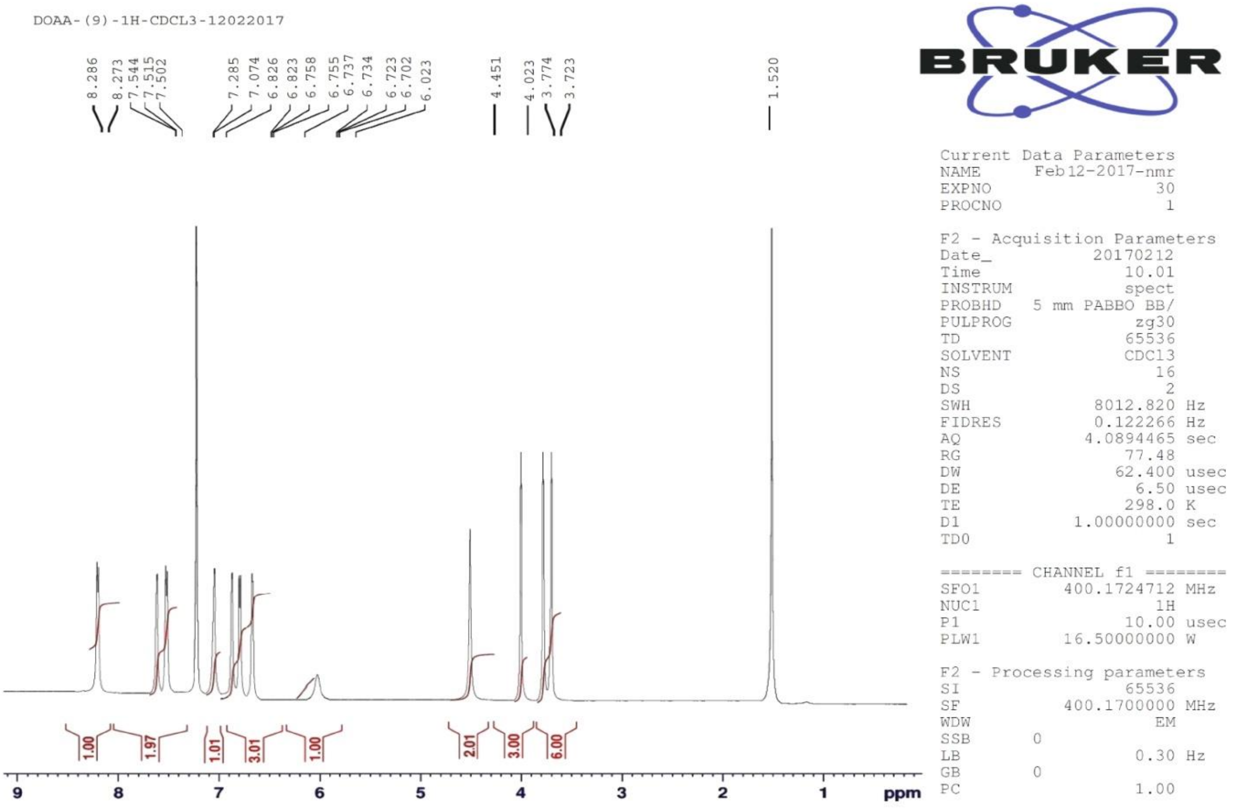

S13: ^{1}H NMR spectrum of metabolite-**3** (400 MHz, $CDCl_3$).

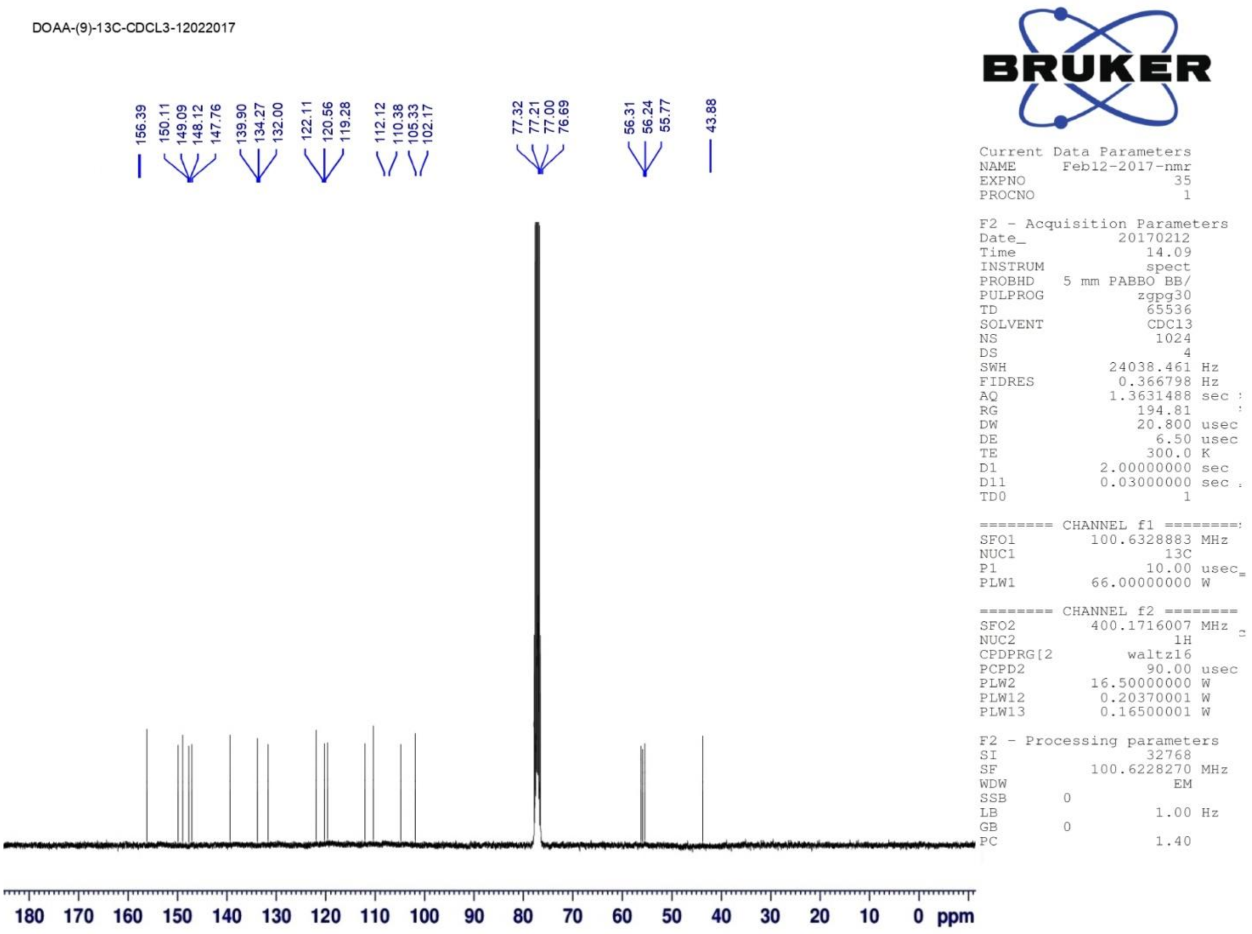

S14: ^{13}C NMR spectrum of metabolite-**3** (100 MHz, $CDCl_3$).

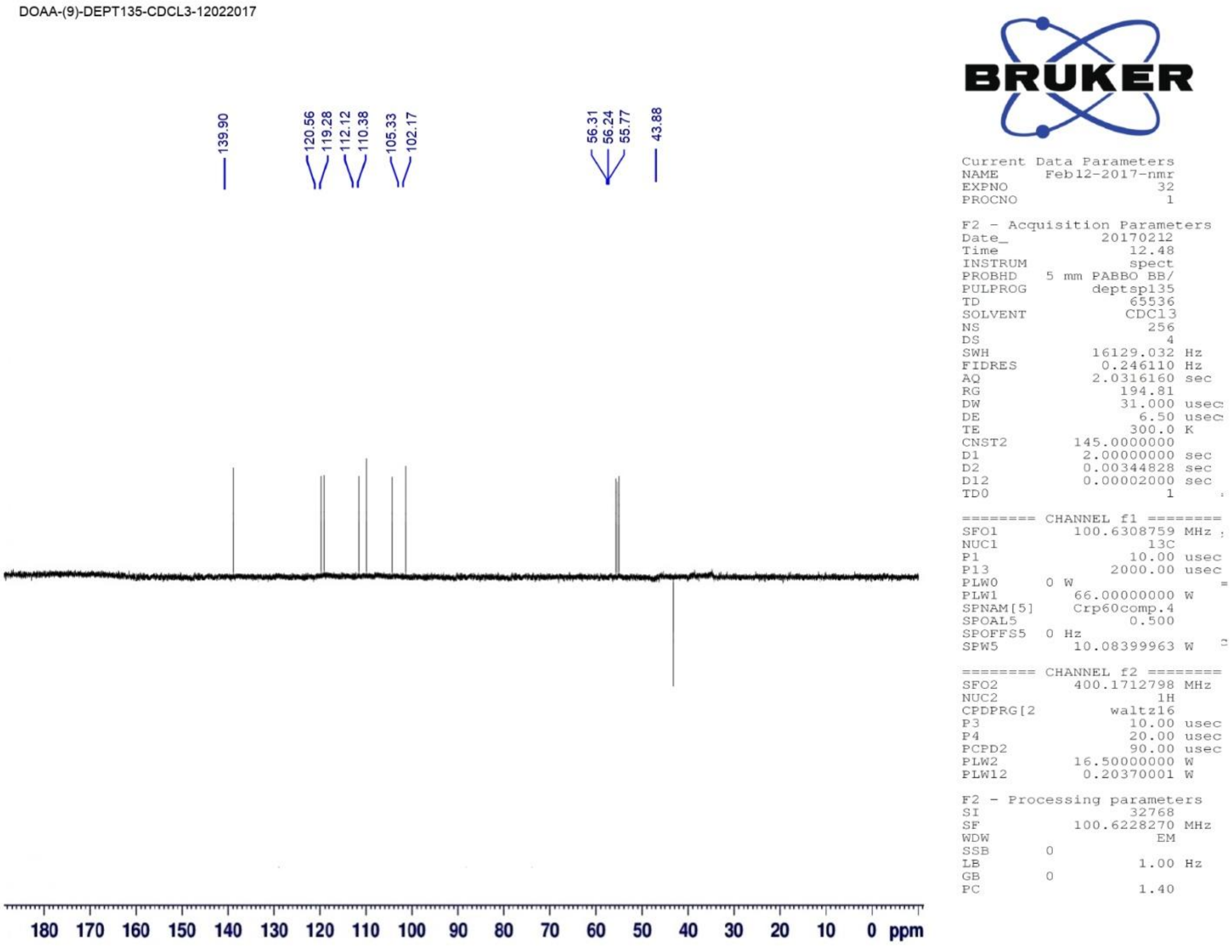

S15: DEPT135 spectrum of metabolite-**3** (100 MHz, $CDCl_3$).

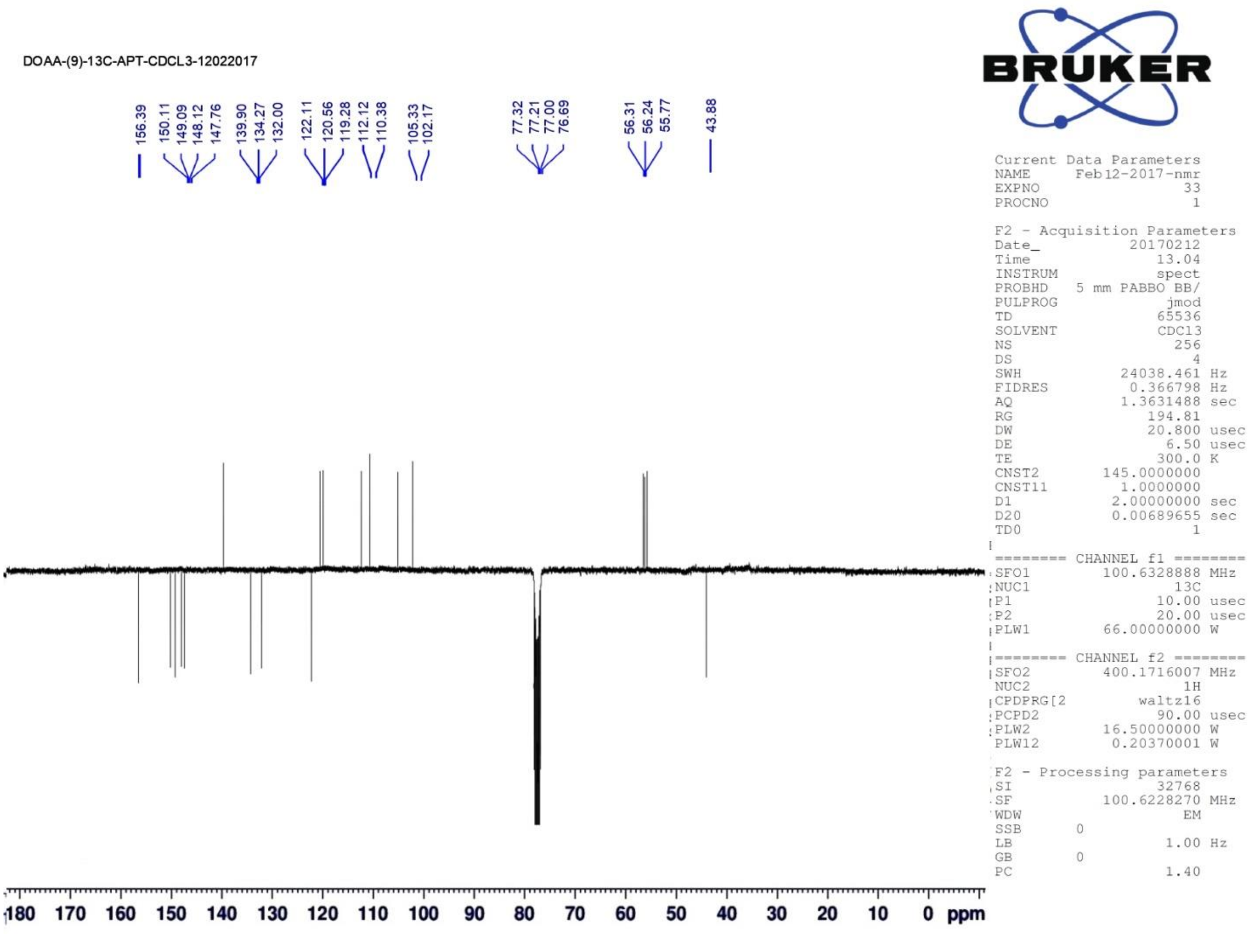

S16: APT spectrum of metabolite-**3** (100 MHz, $CDCl_3$).

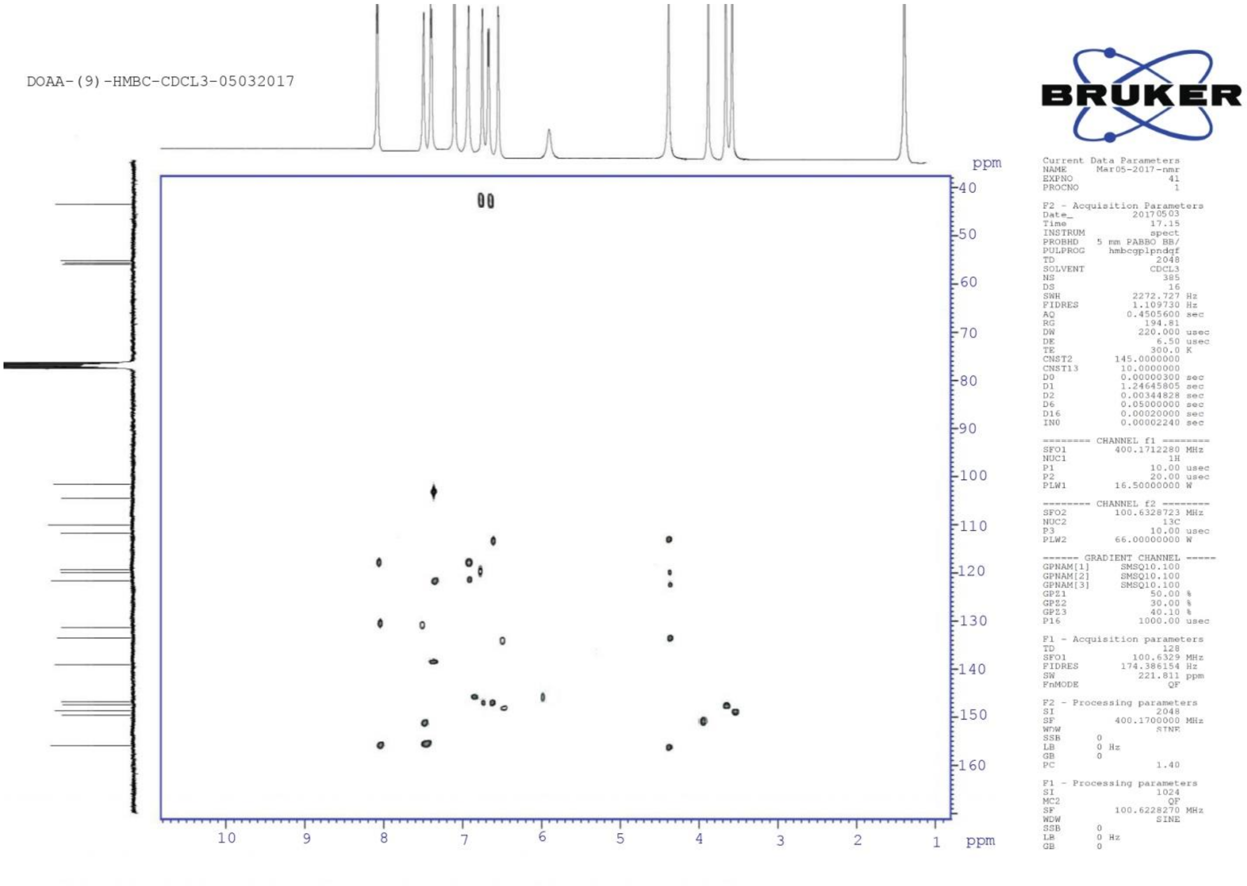

S17: HMBC NMR spectrum of metabolite-**3** ($CDCl_3$).

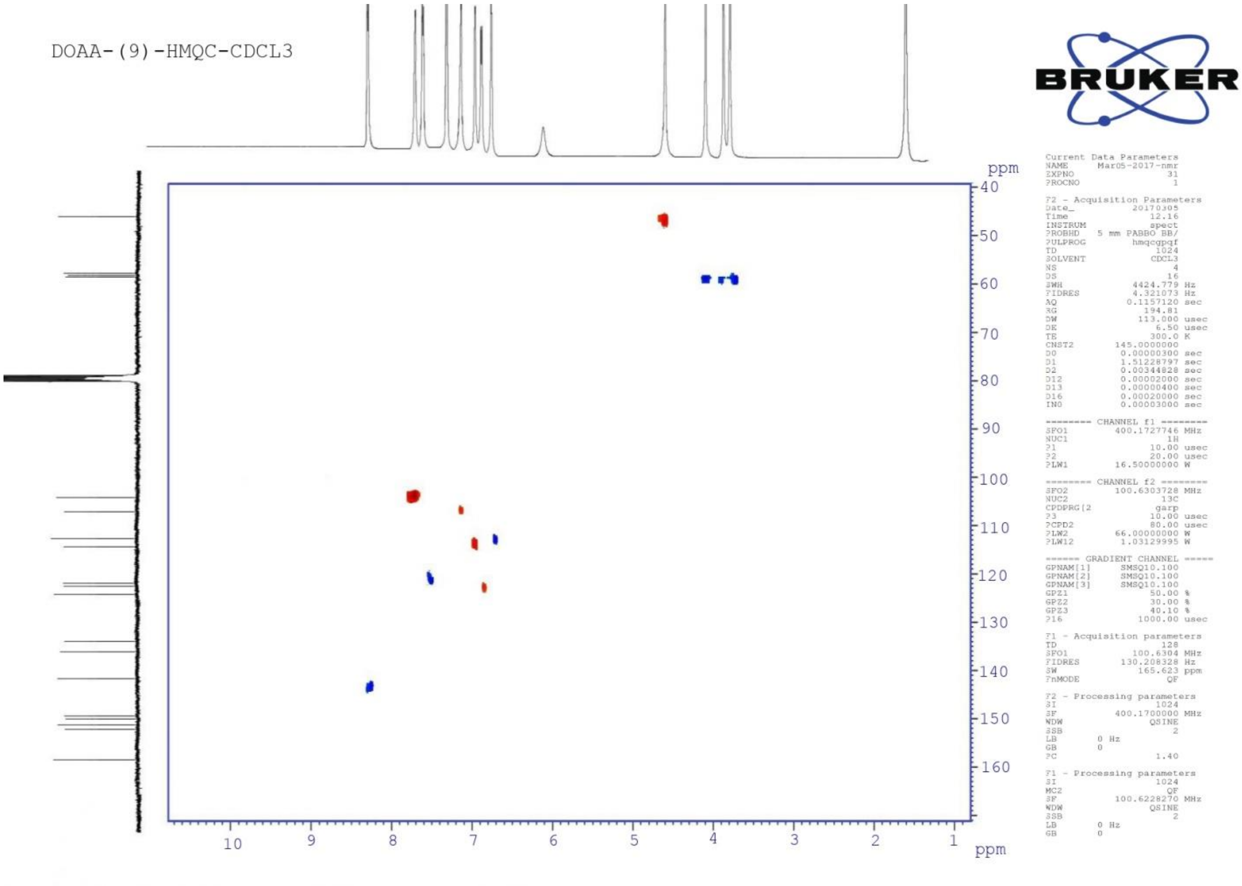

S18: HMQC NMR spectrum of metabolite-**3** ($CDCl_3$).

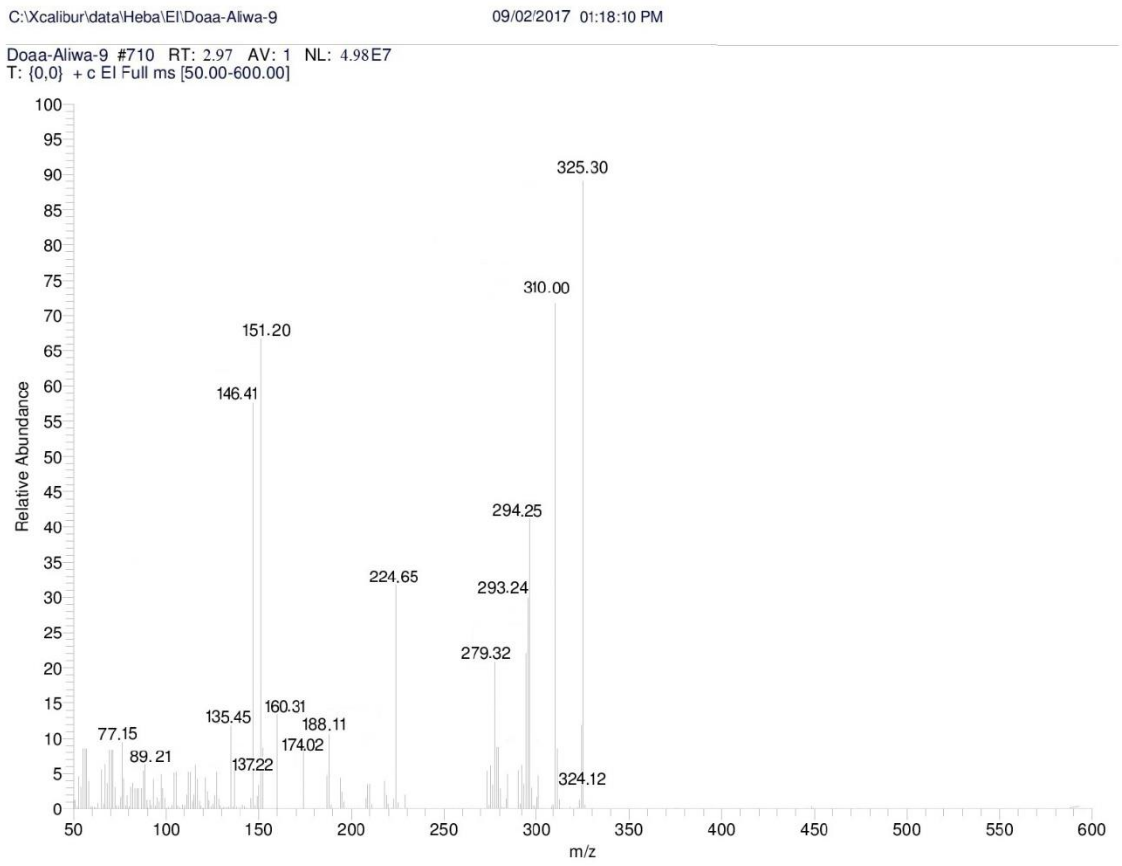

S19: Mass spectrum of metabolite**-3.**

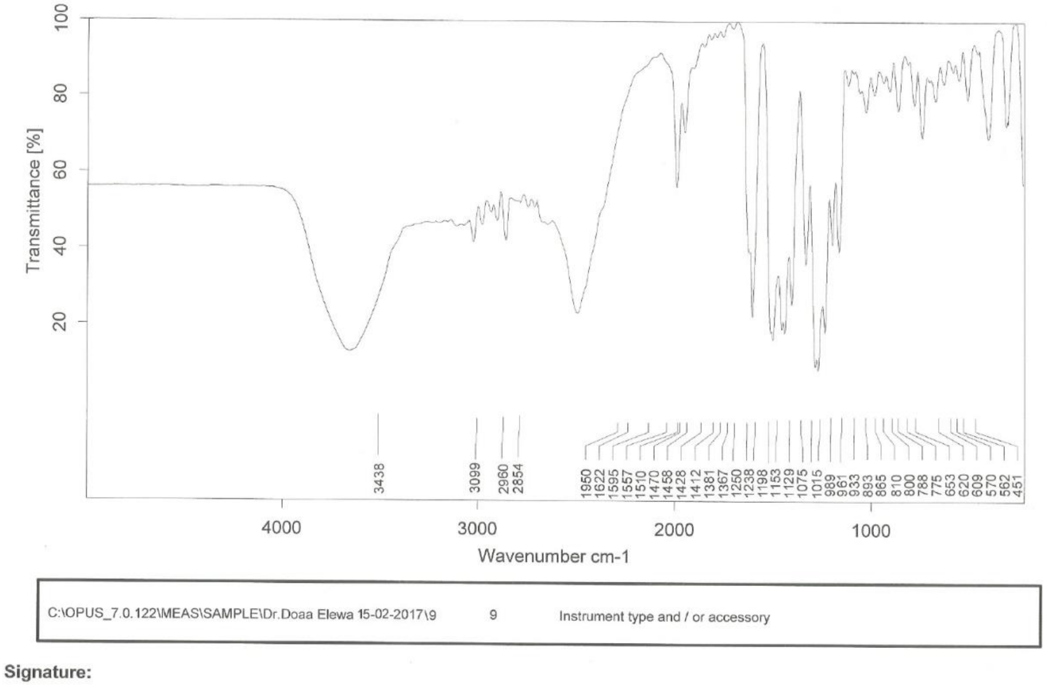

S20: IR of metabolite-**3**.

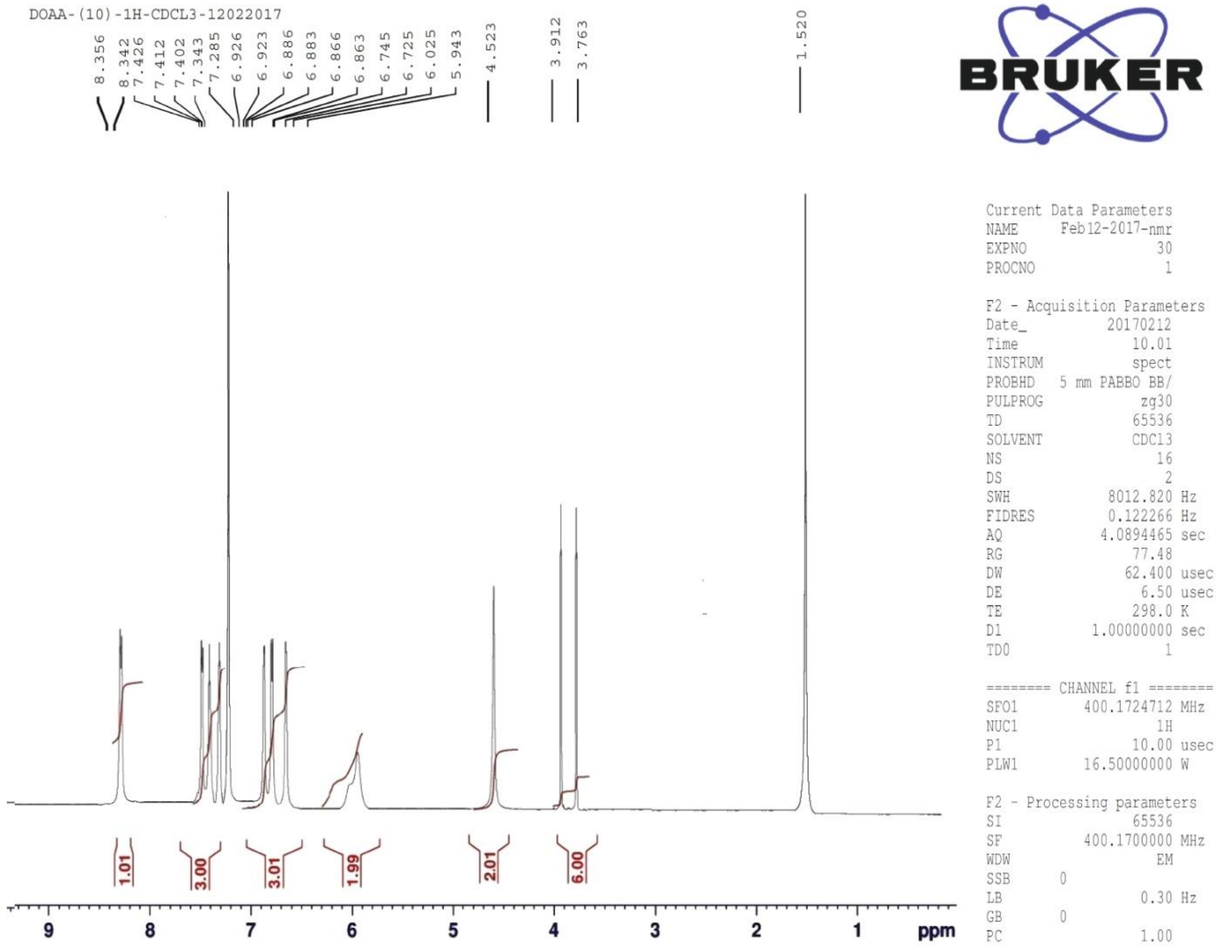

S21: ^{1}H NMR spectrum of metabolite-**4** (400 MHz, $CDCl_3$).

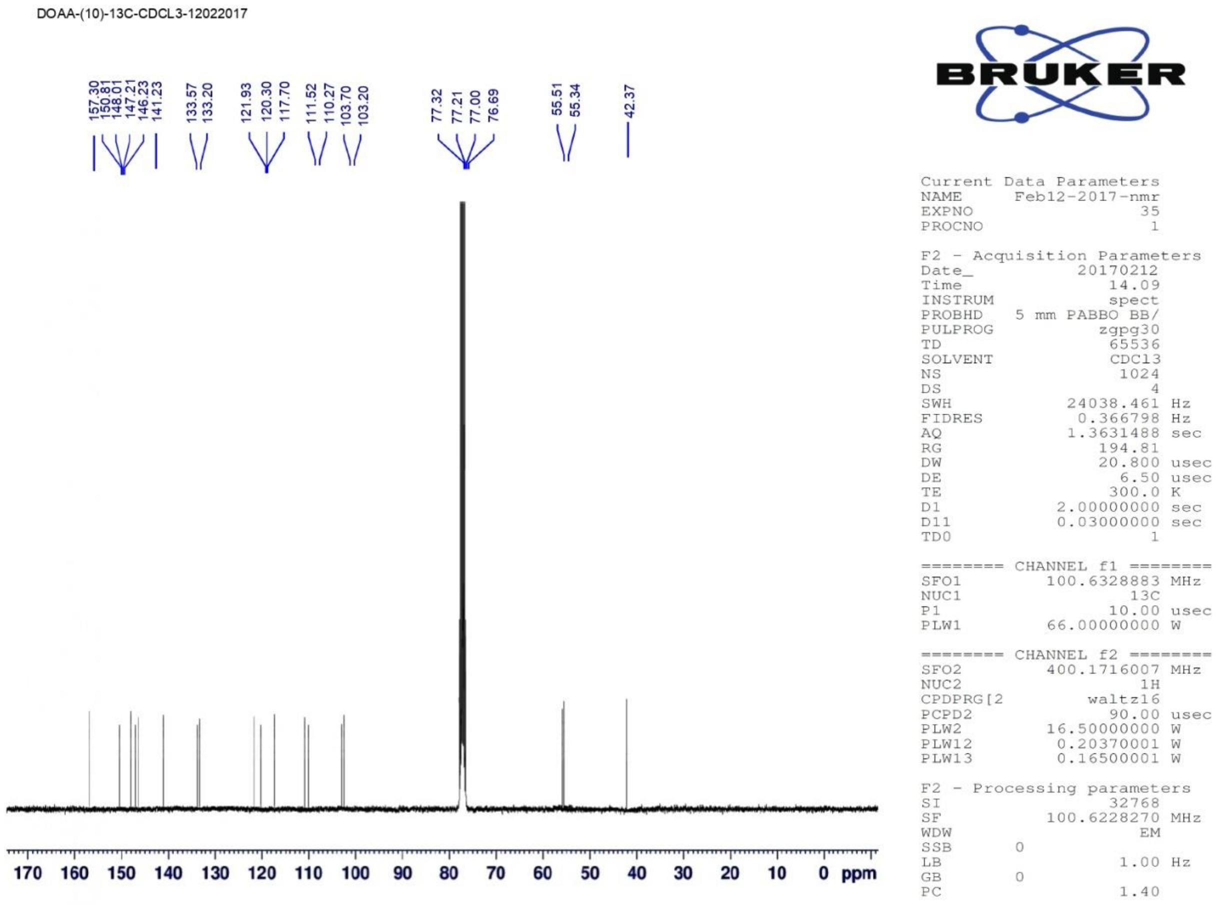

S22: ^{13}C NMR spectrum of metabolite-**4** (100 MHz, $CDCl_3$).

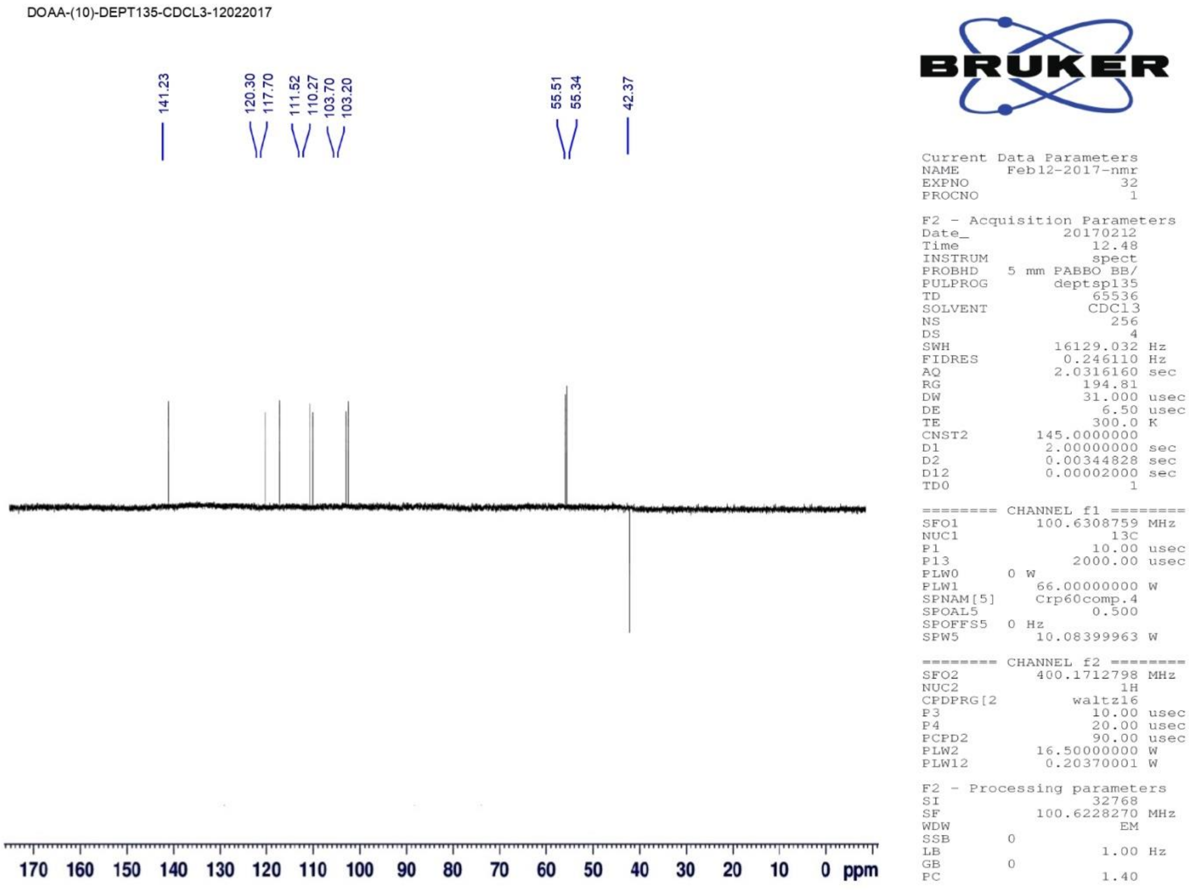

S23: DEPT135 spectrum of metabolite-**4** (100 MHz, $CDCl_3$).

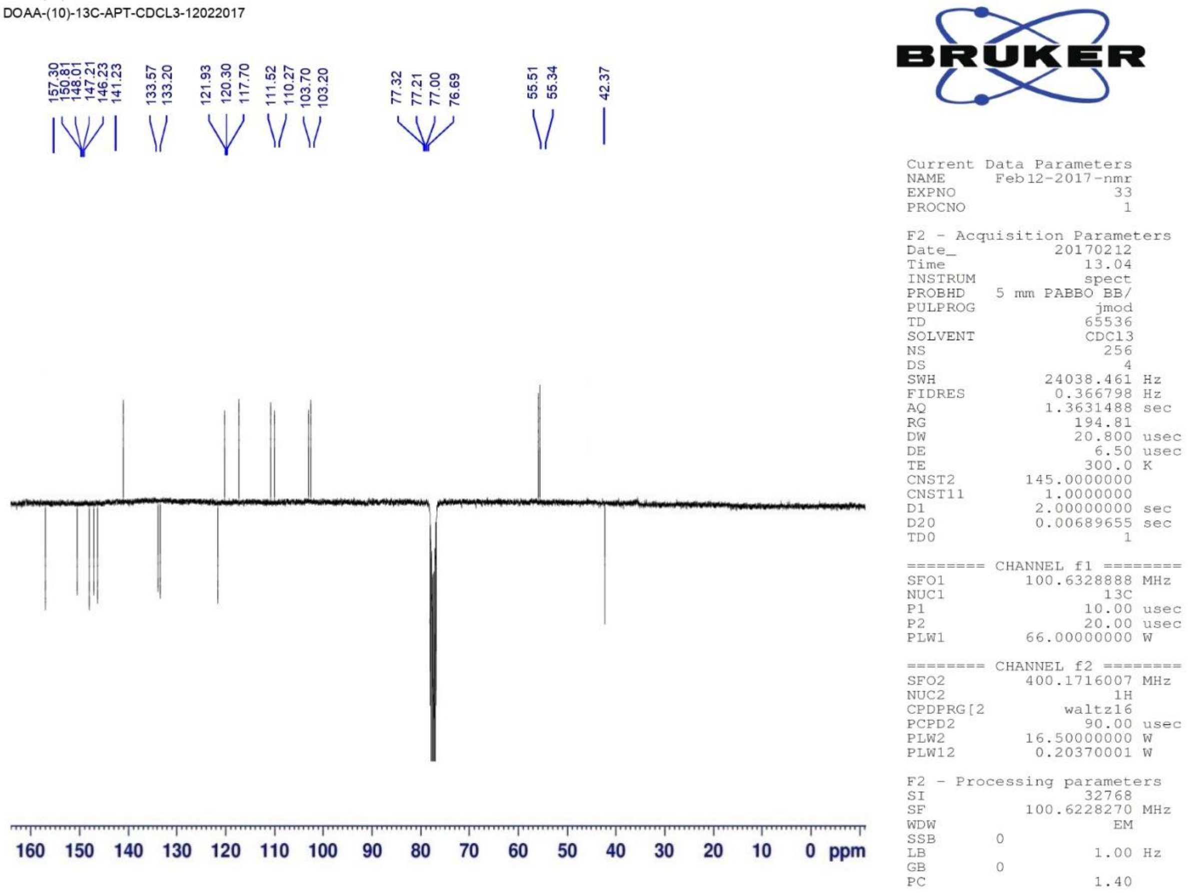

S24: APT spectrum of metabolite-**4** (100 MHz, $CDCl_3$).

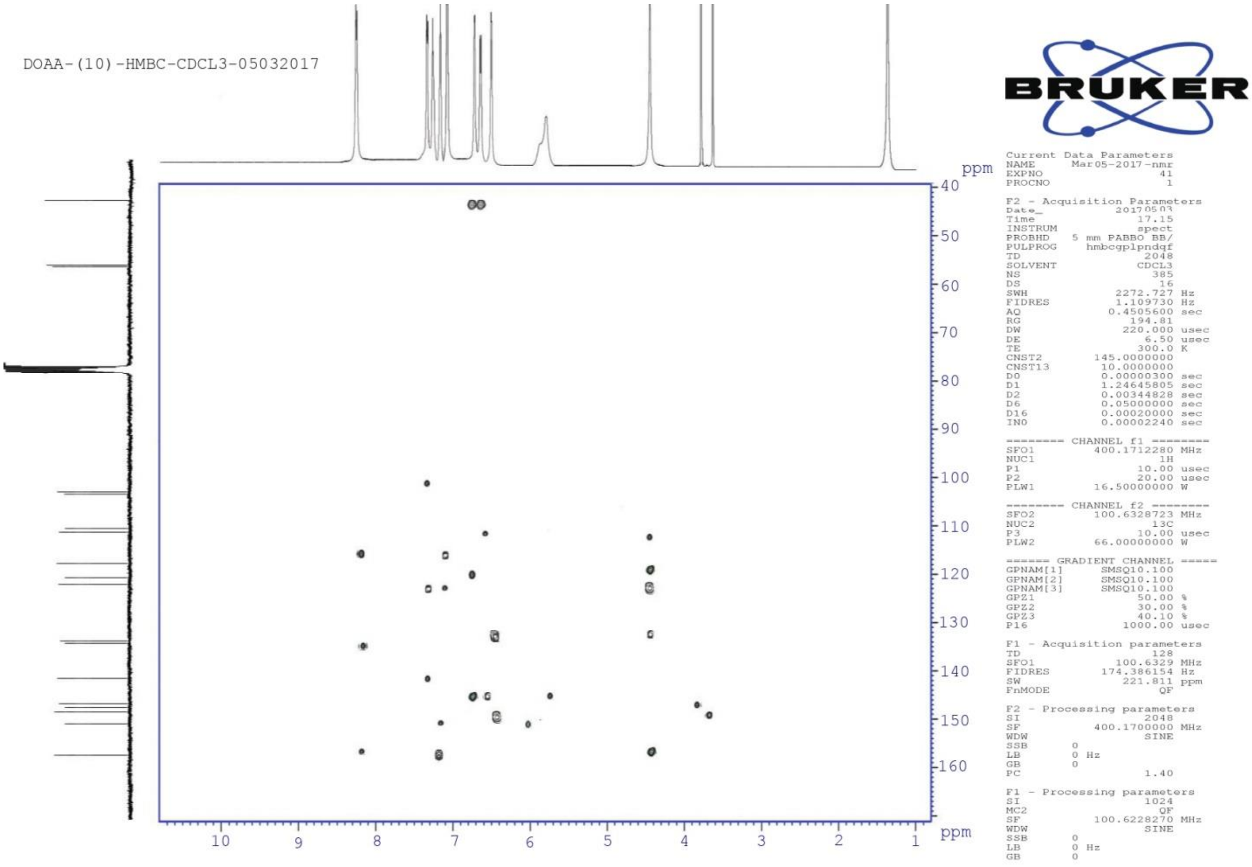

S25: HMBC NMR spectrum of metabolite-**4** ($CDCl_3$).

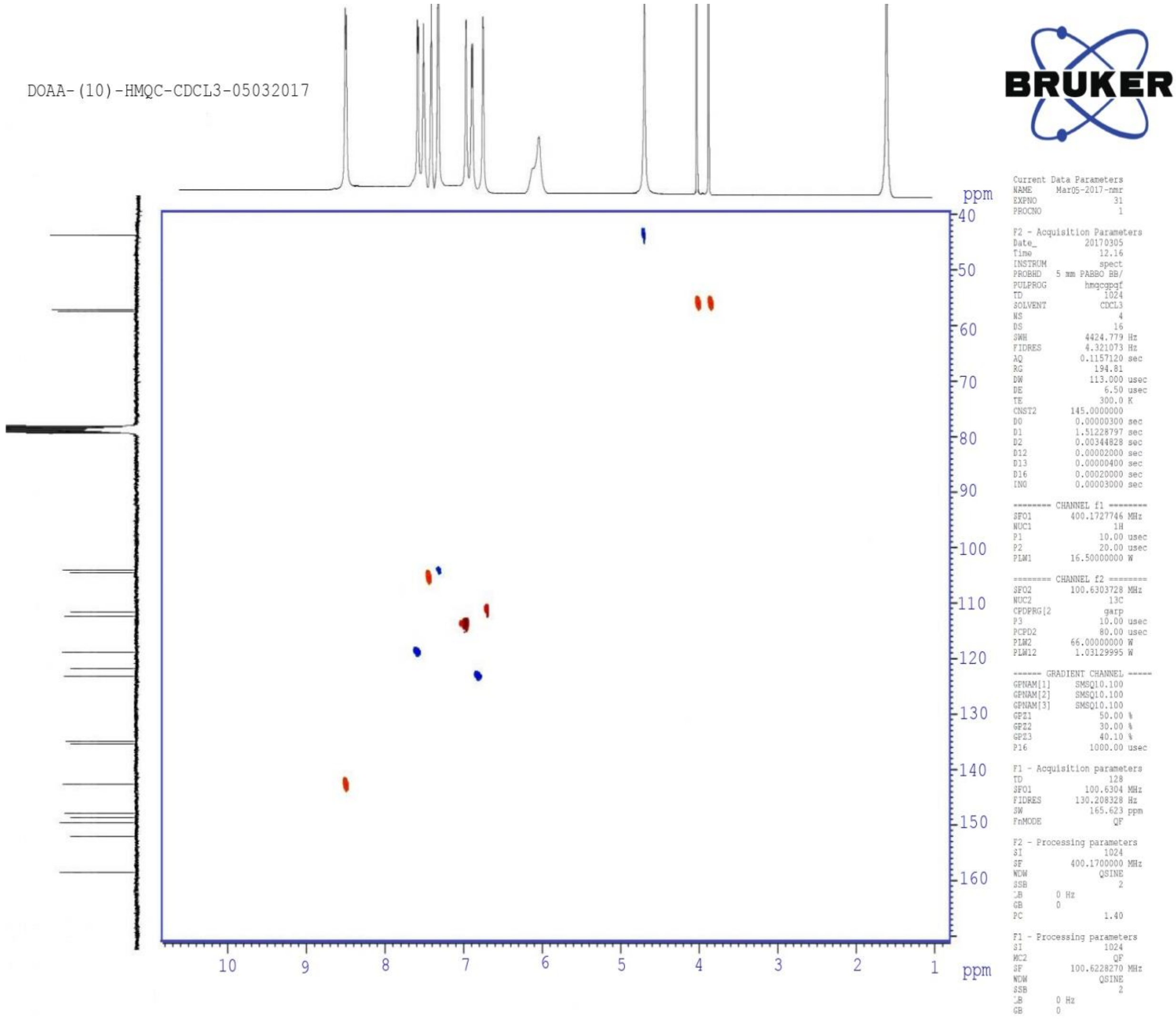

S26: HMQC NMR spectrum of metabolite-**4** ($CDCl_3$).

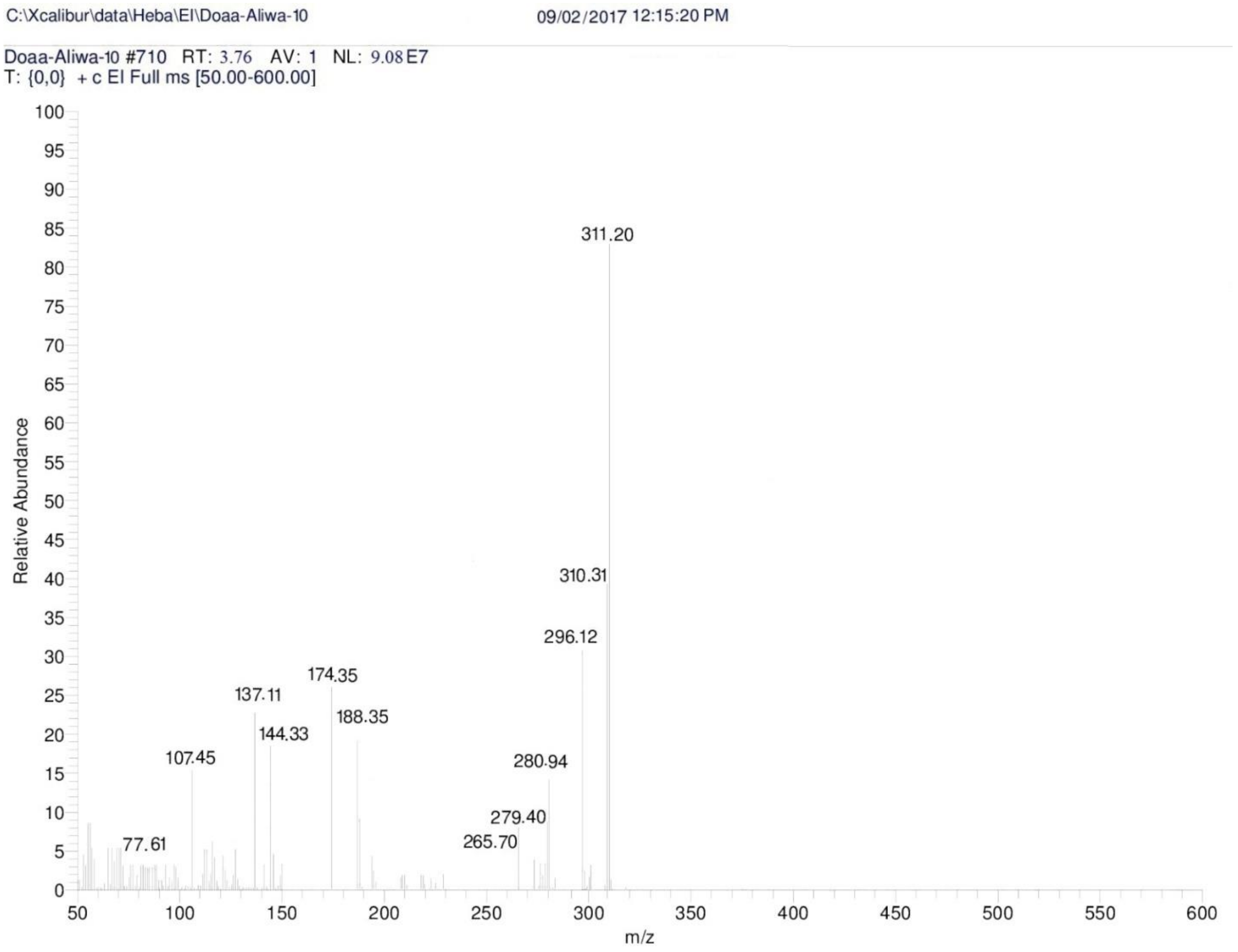

S27: Mass spectrum of metabolite**-4.**

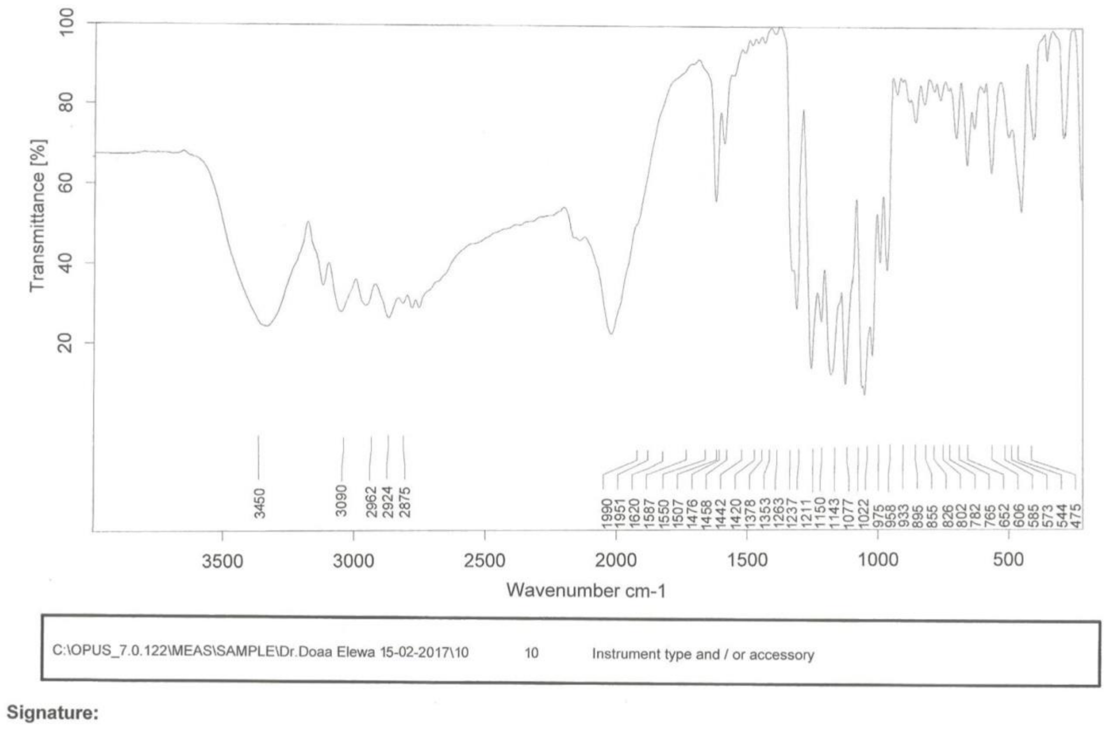

S28: IR of metabolite-**4**

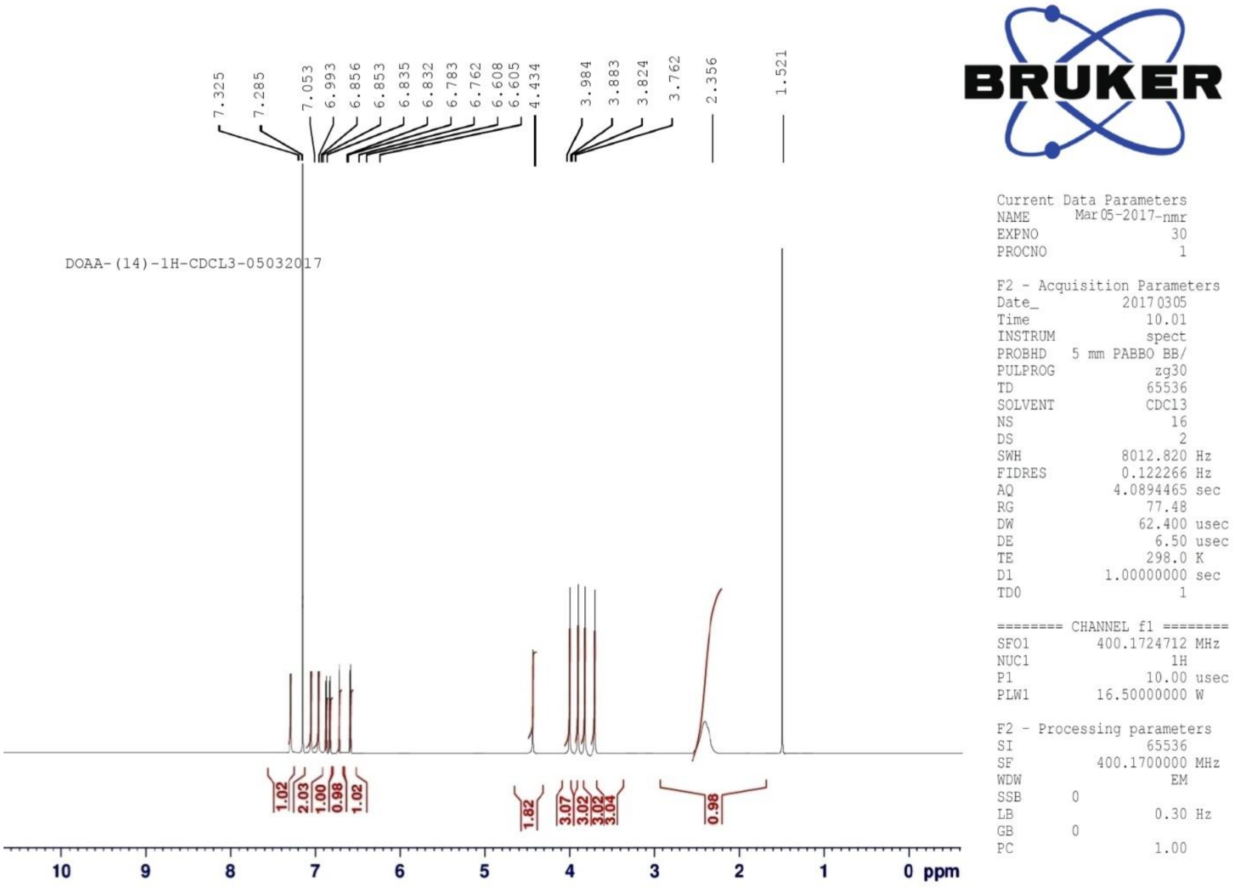

S29: ^{1}H NMR spectrum of metabolite-**5** (400 MHz, $CDCl_3$).

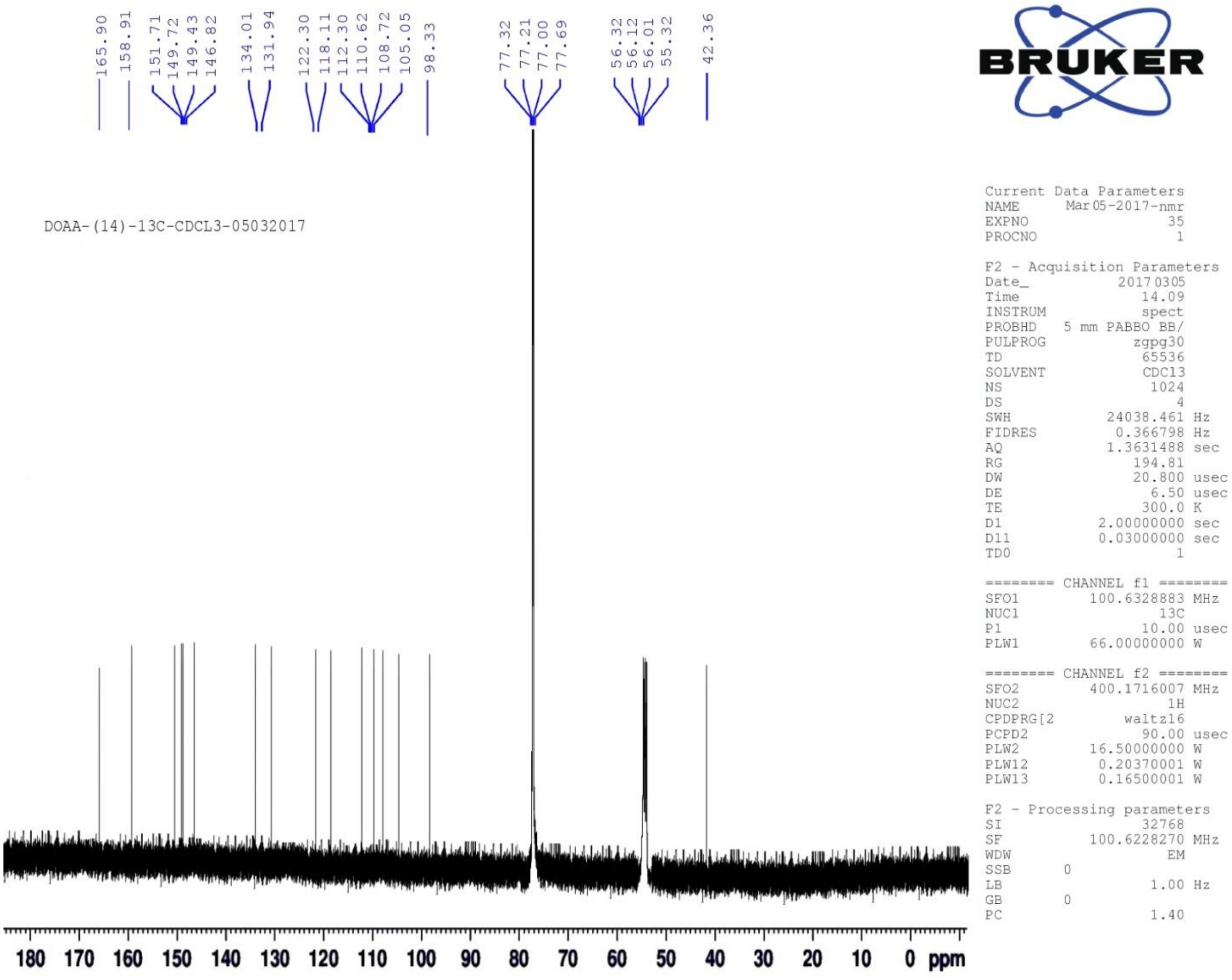

S30: ^{13}C NMR spectrum of metabolite-**5** (100 MHz, $CDCl_3$).

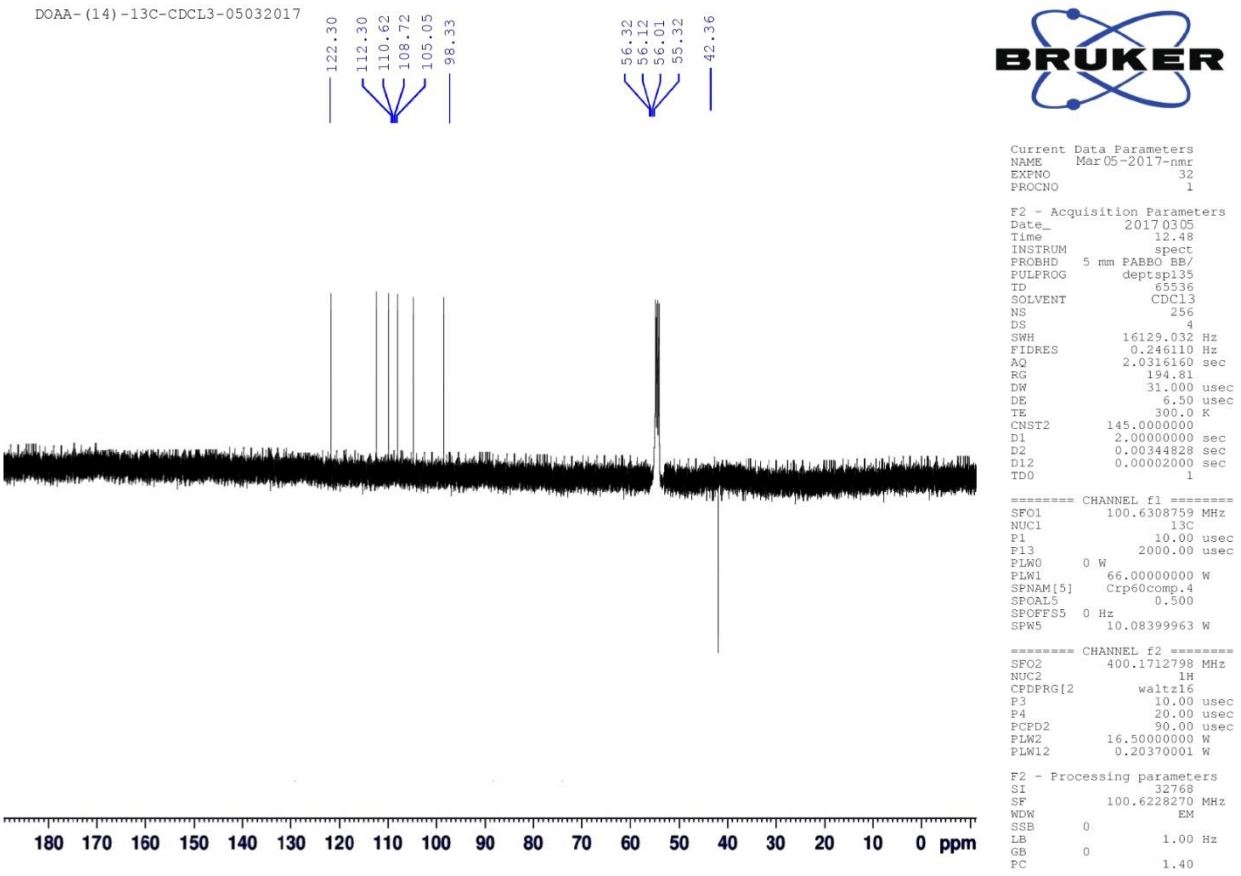

S31: DEPT 135 spectrum of metabolite-**5** (100 MHz, $CDCl_3$).

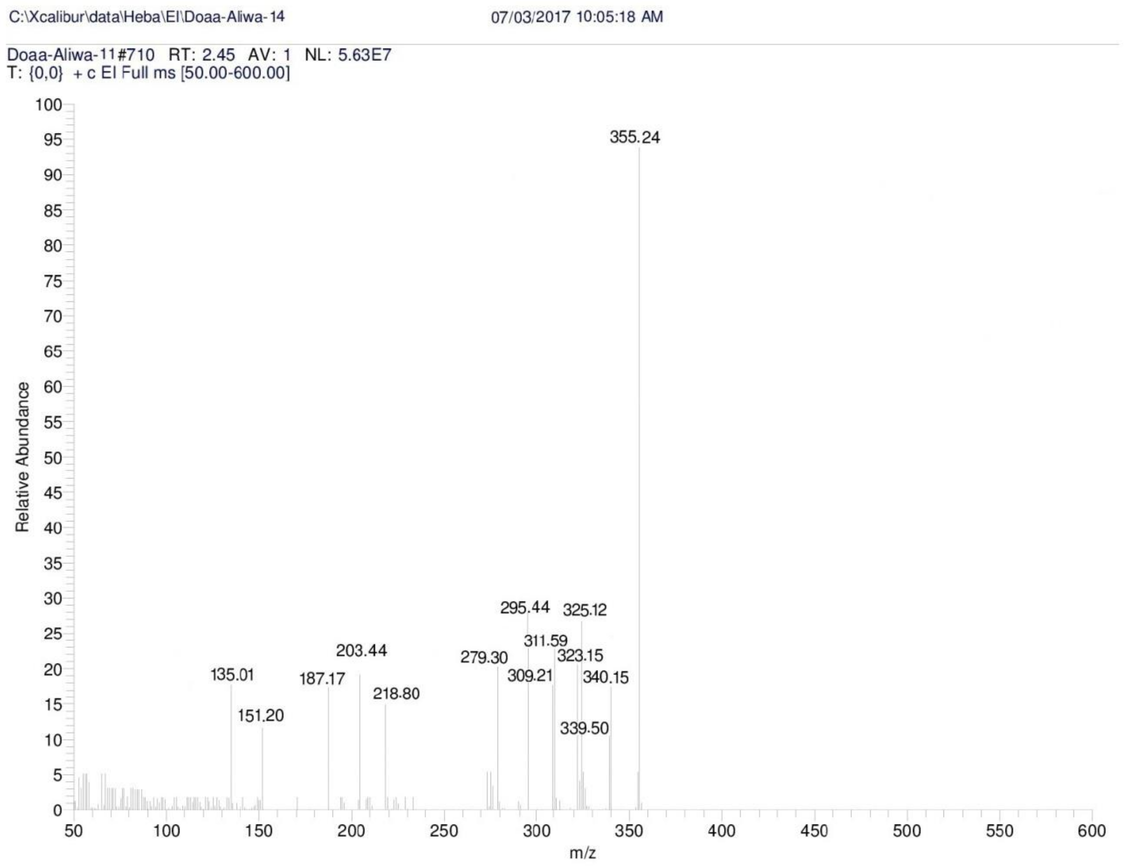

S32: Mass spectrum of metabolite-**5.**

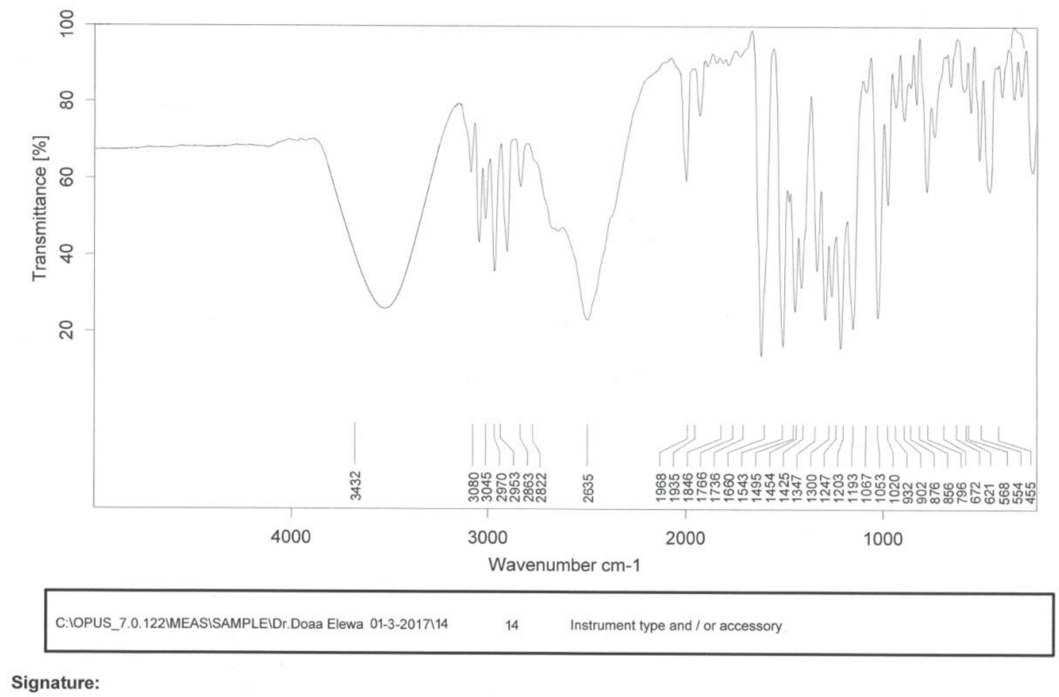

S33: IR spectrum of metabolite-**5.**

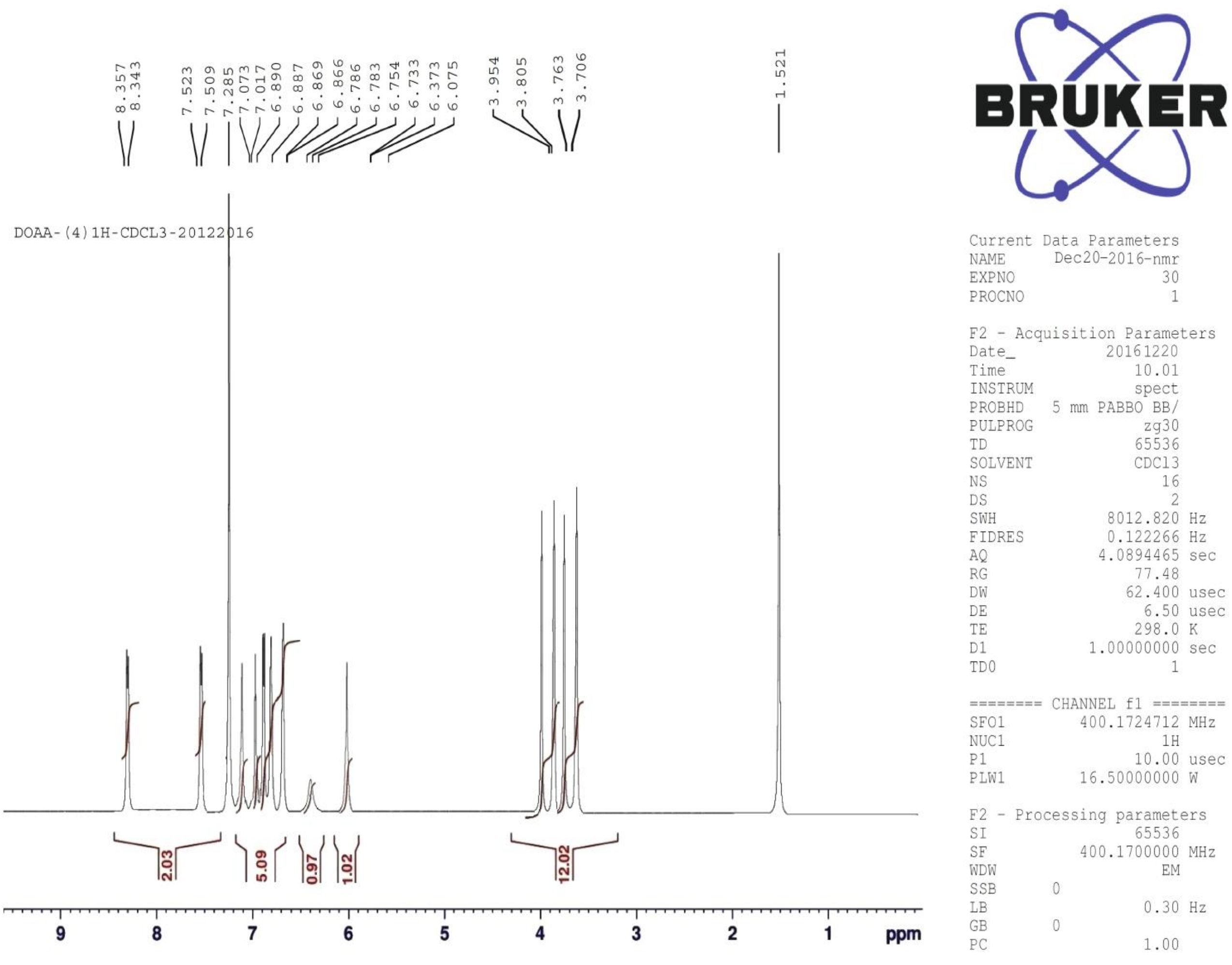

S34: ^{1}H NMR spectrum of metabolite-**6** (400 MHz, $CDCl_3$).

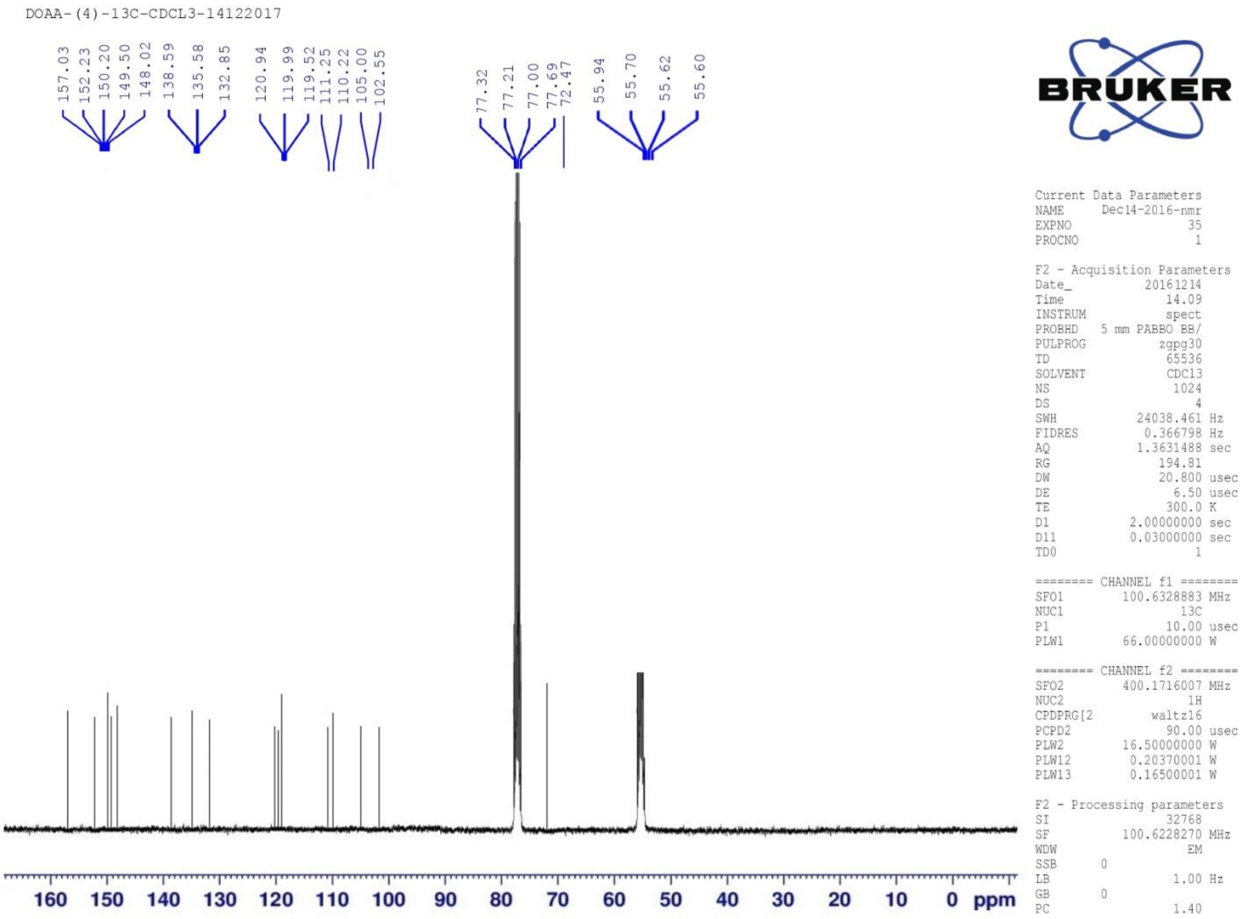

S35: ^{13}C NMR spectrum of metabolite-**6** (100 MHz, $CDCl_3$).

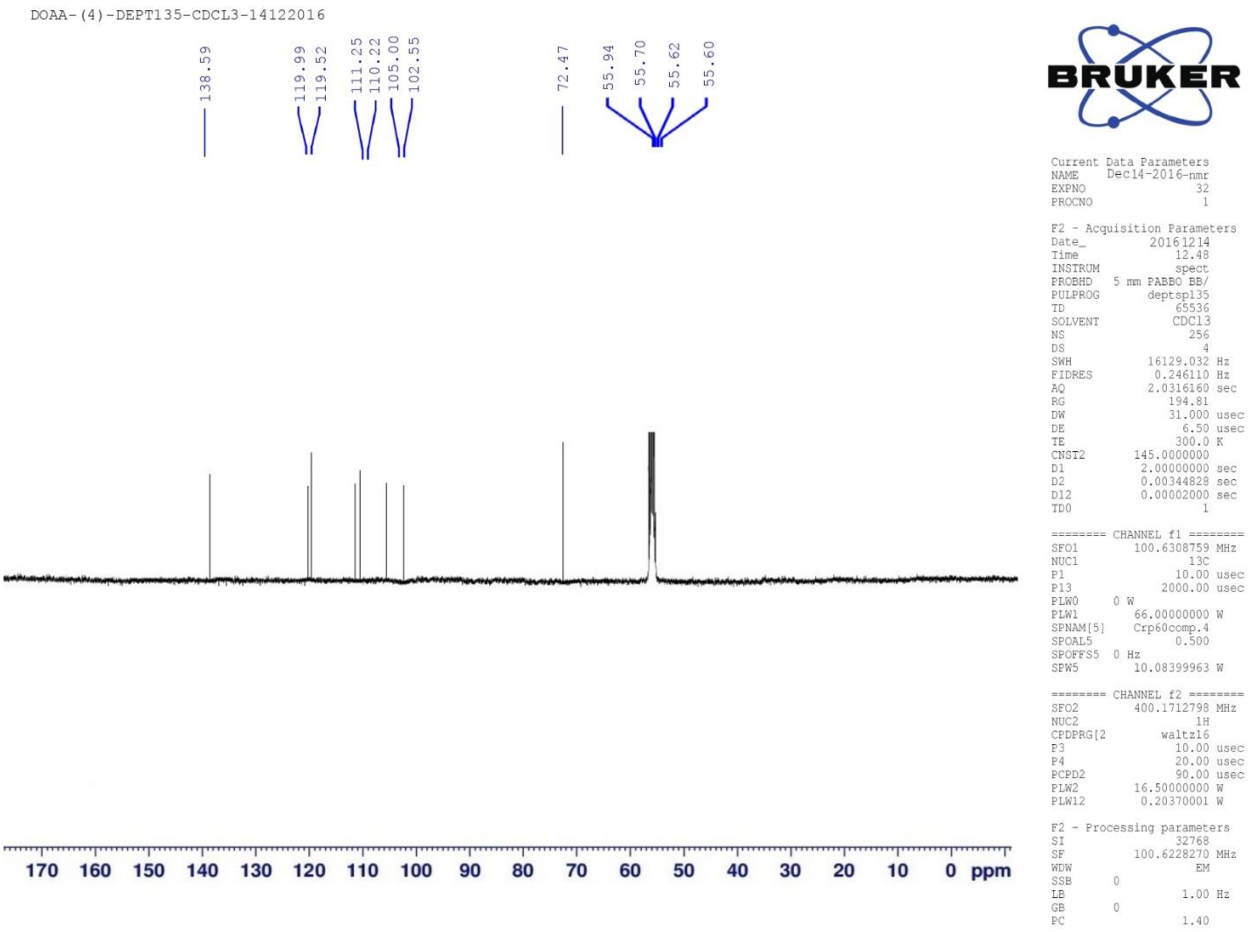

S36: DEPT 135 spectrum of metabolite-**6** (100 MHz, $CDCl_3$).

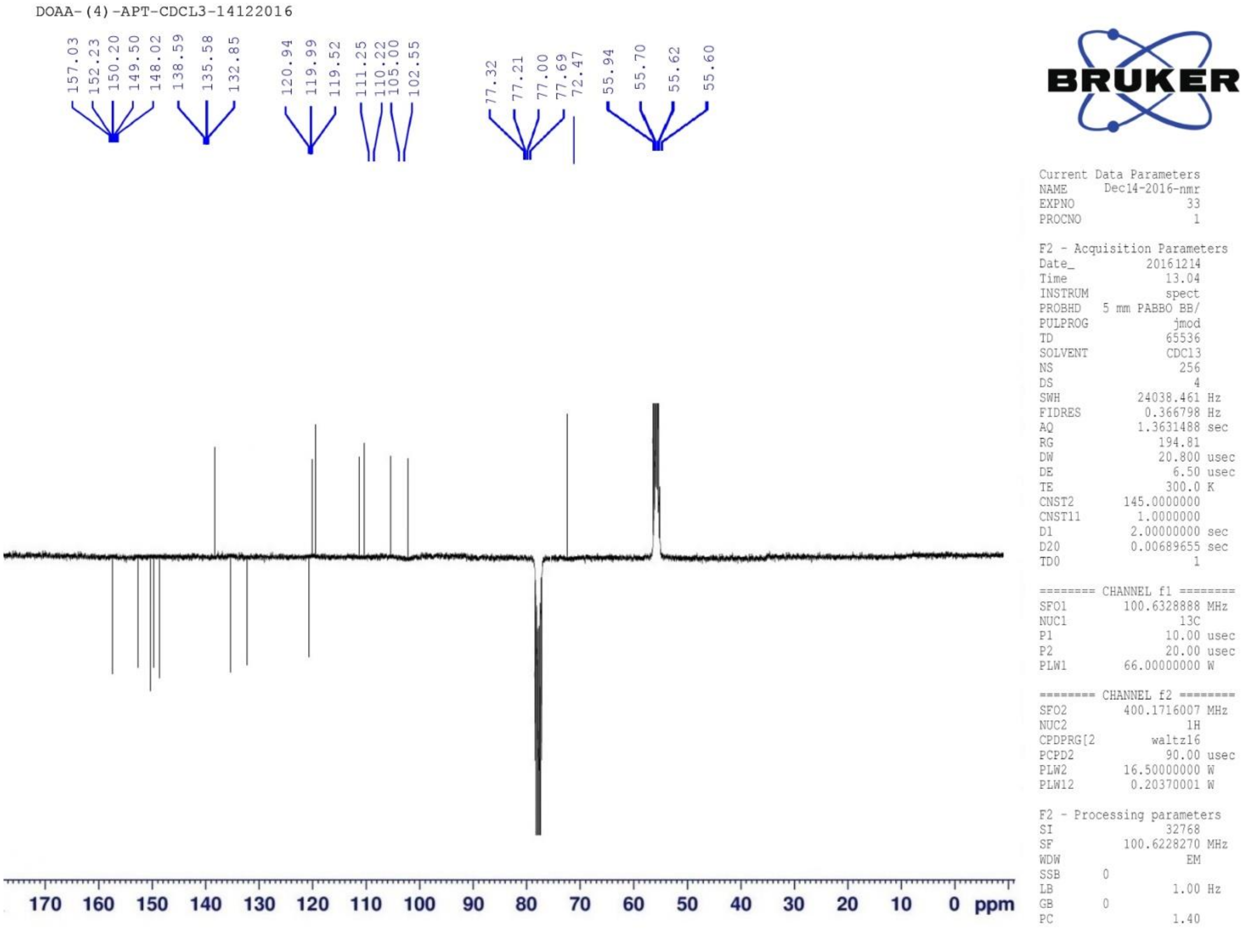

S37: APT NMR spectrum of metabolite-**6** ($CDCl_3$).

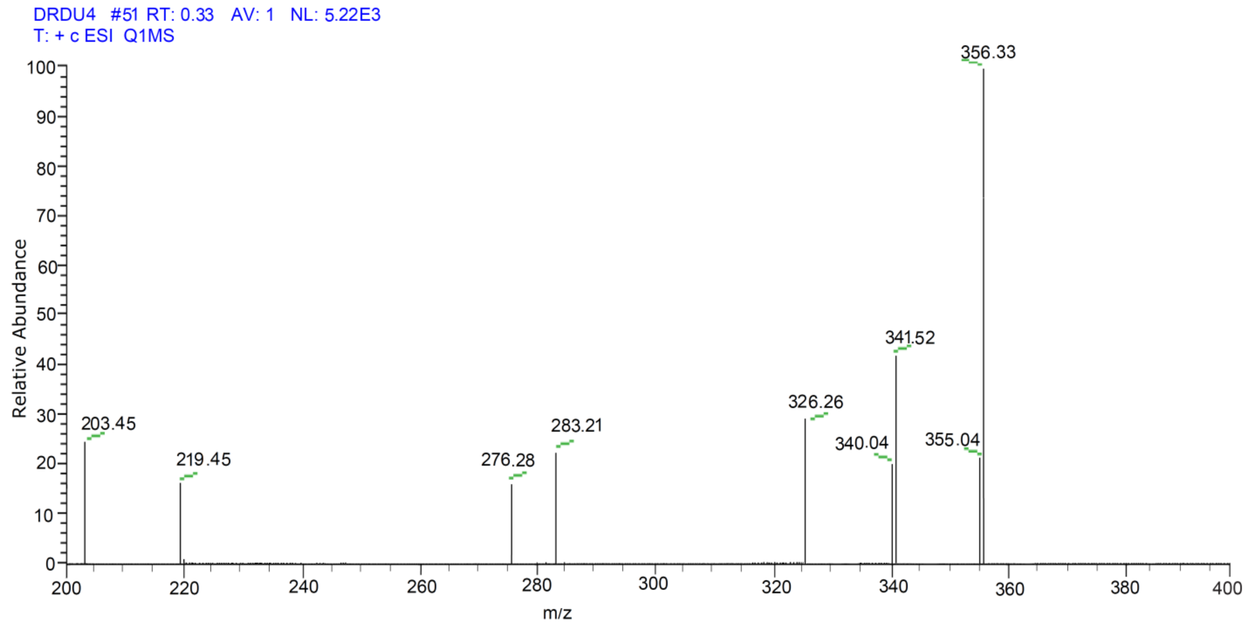

S38: (+) ESI-MS analysis of metabolite**-6.**

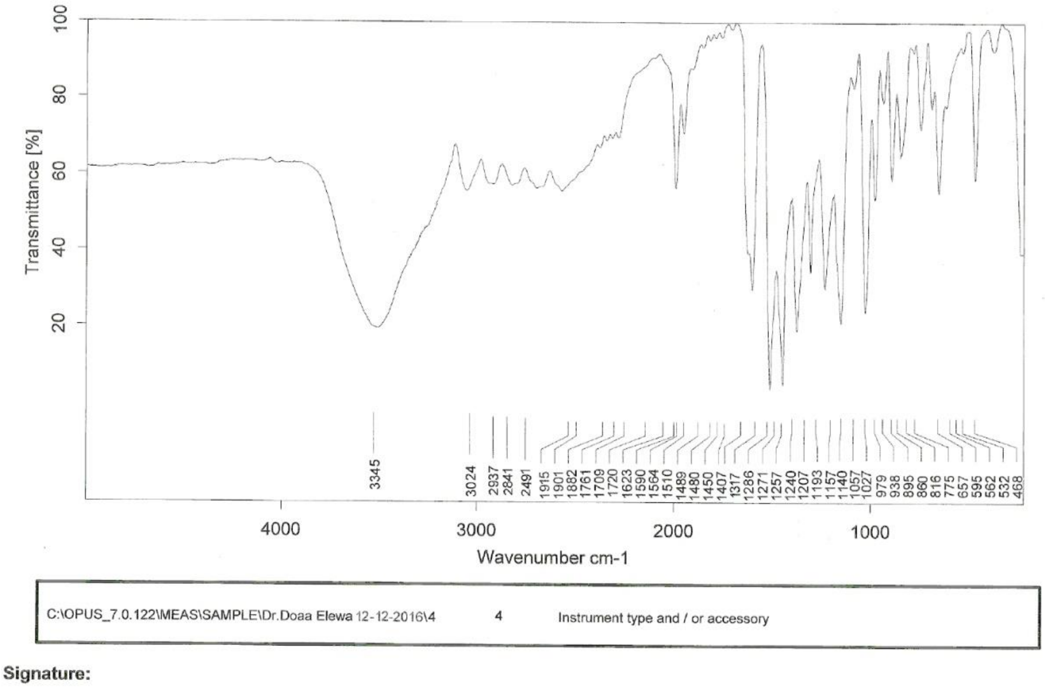

S39: IR spectrum of metabolite-**6.**

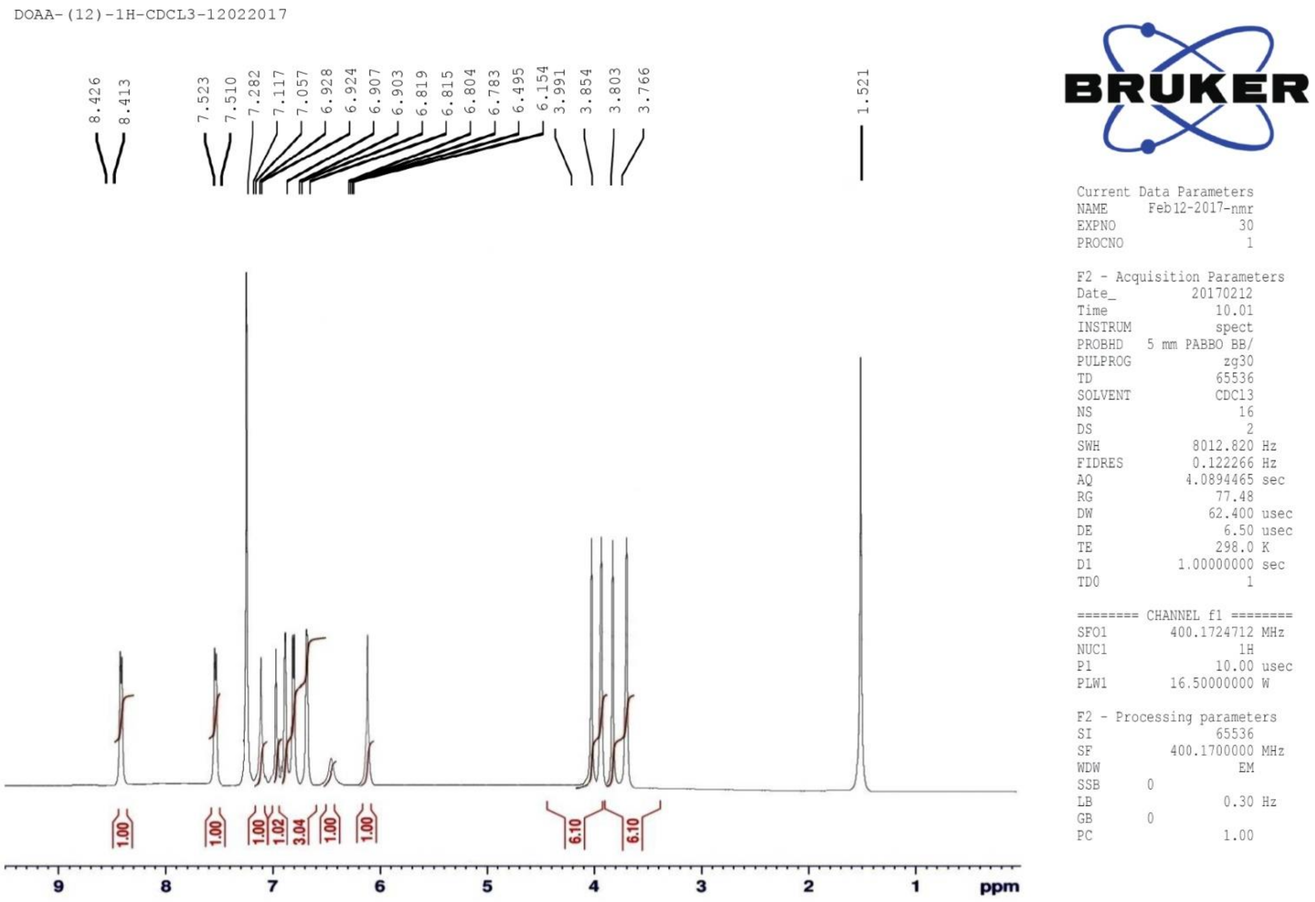

S40: ^{1}H NMR spectrum of metabolite-**7** (400 MHz, $CDCl_3$).

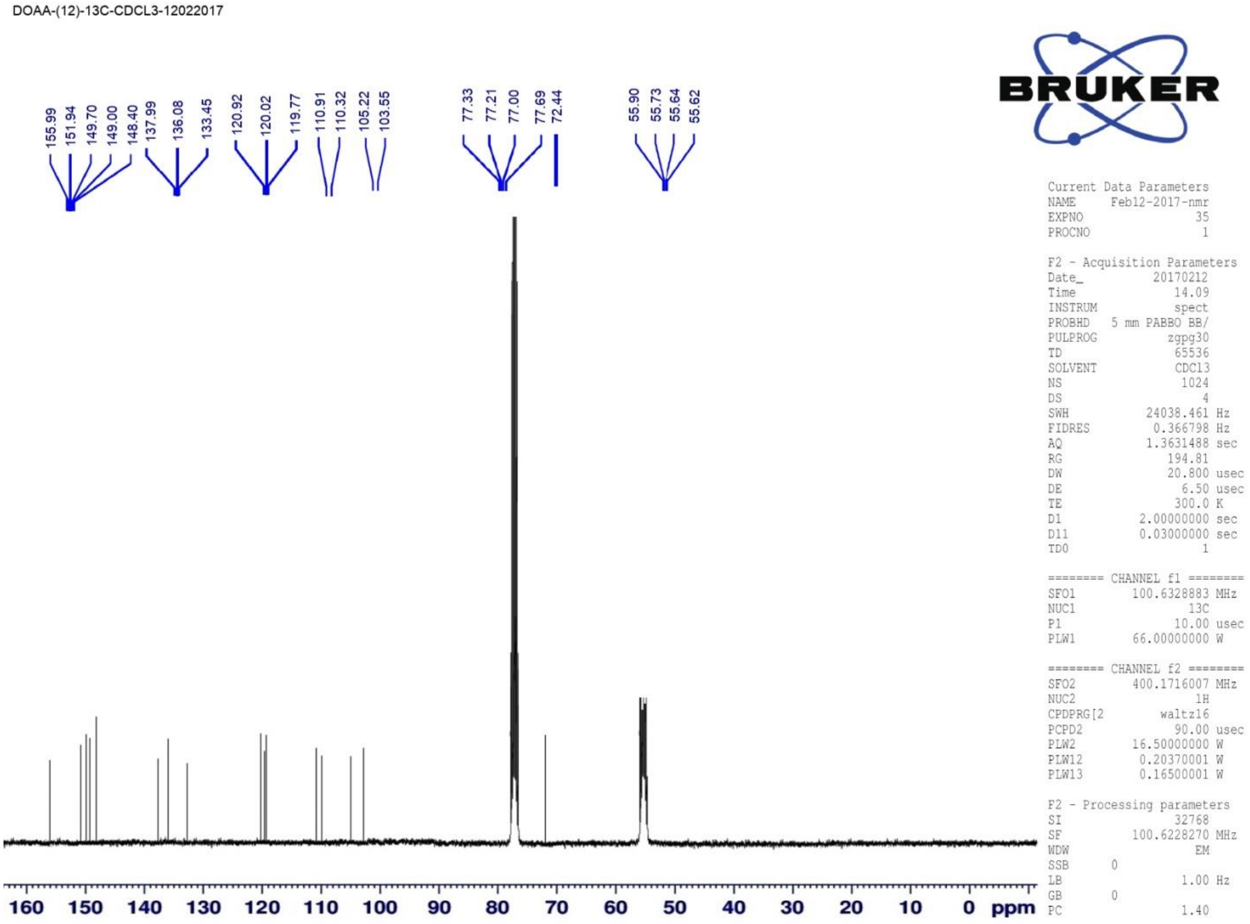

S41: ^{13}C NMR spectrum of metabolite-**7** (100 MHz, $CDCl_3$).

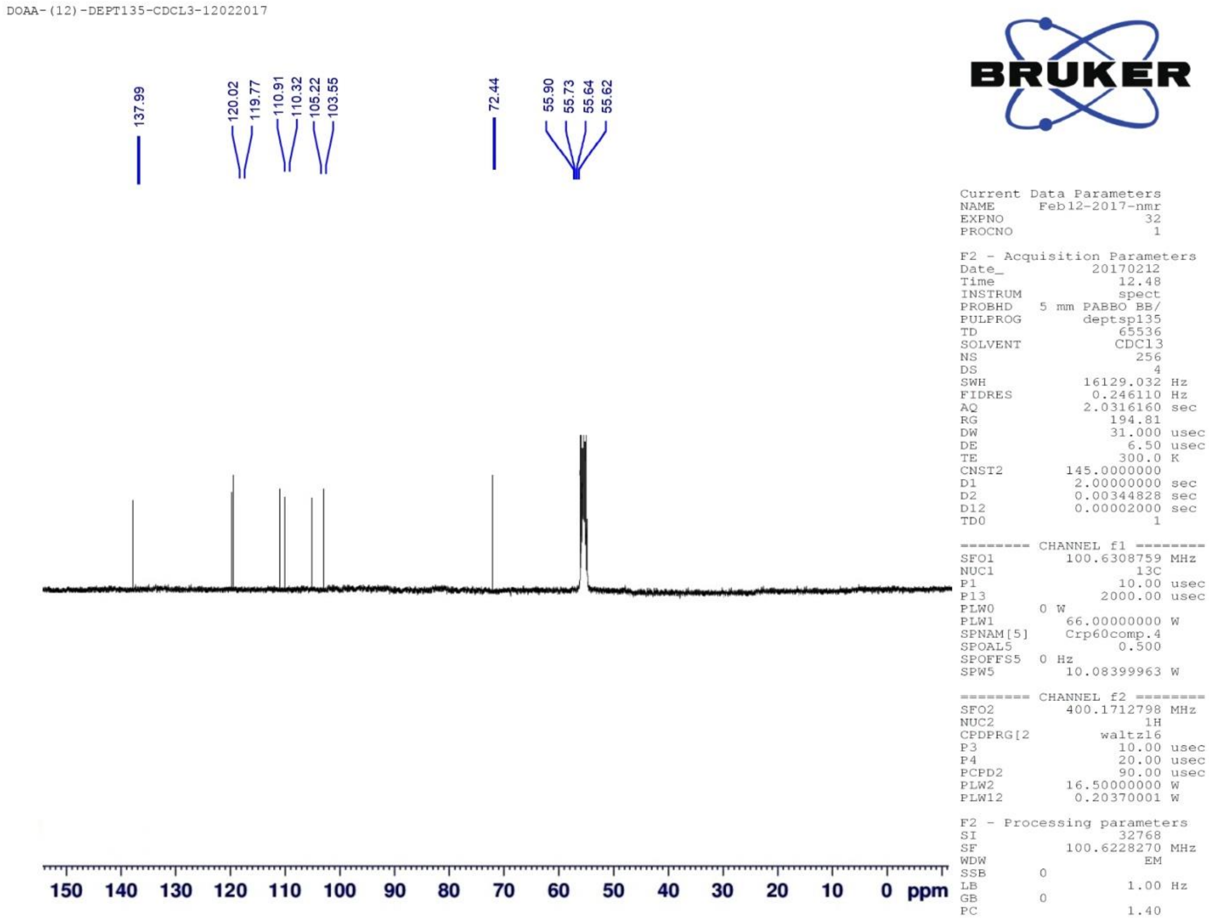

S42: DEPT 135 spectrum of metabolite-**7** (100 MHz, $CDCl_3$).

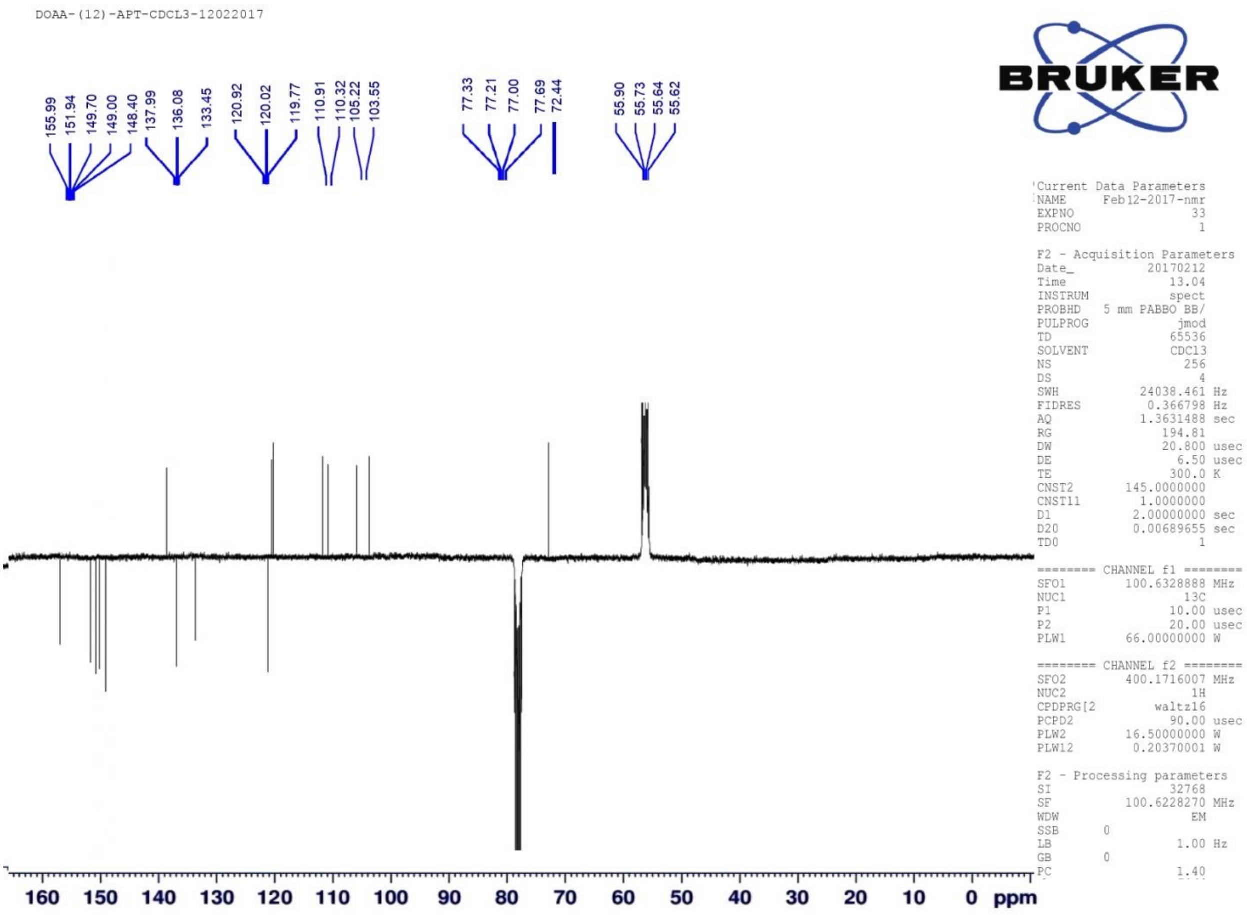

S43: APT spectrum of metabolite-**7** (100 MHz, CDCl3).

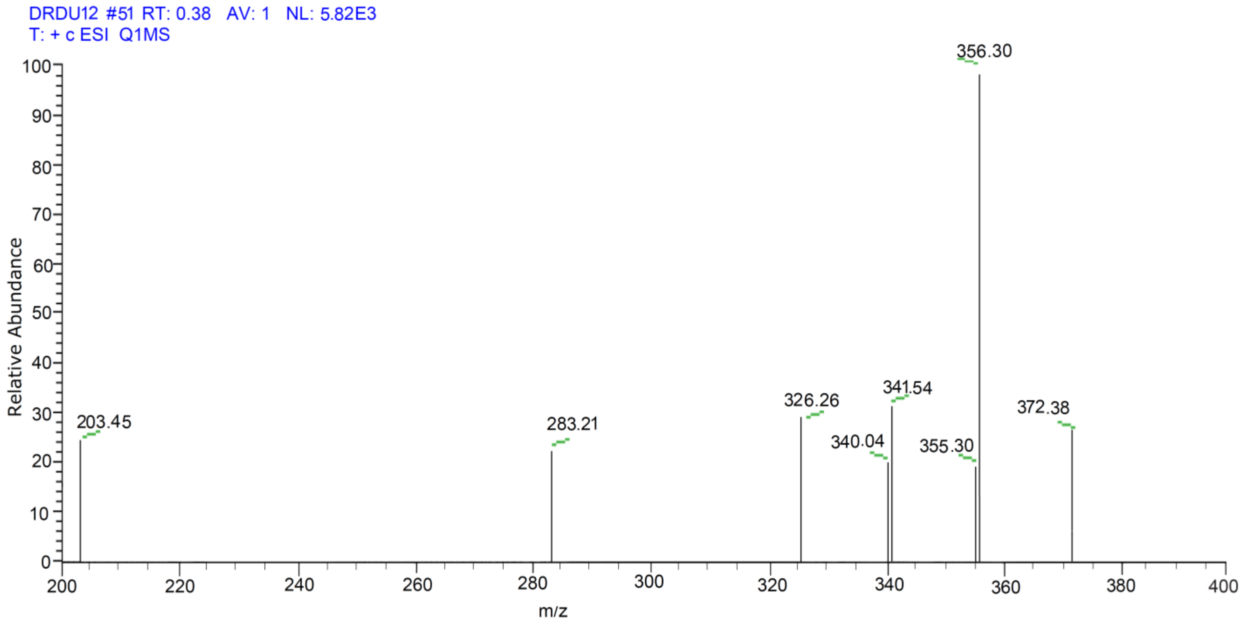

S44: (+) ESI-MS analysis of **7.**

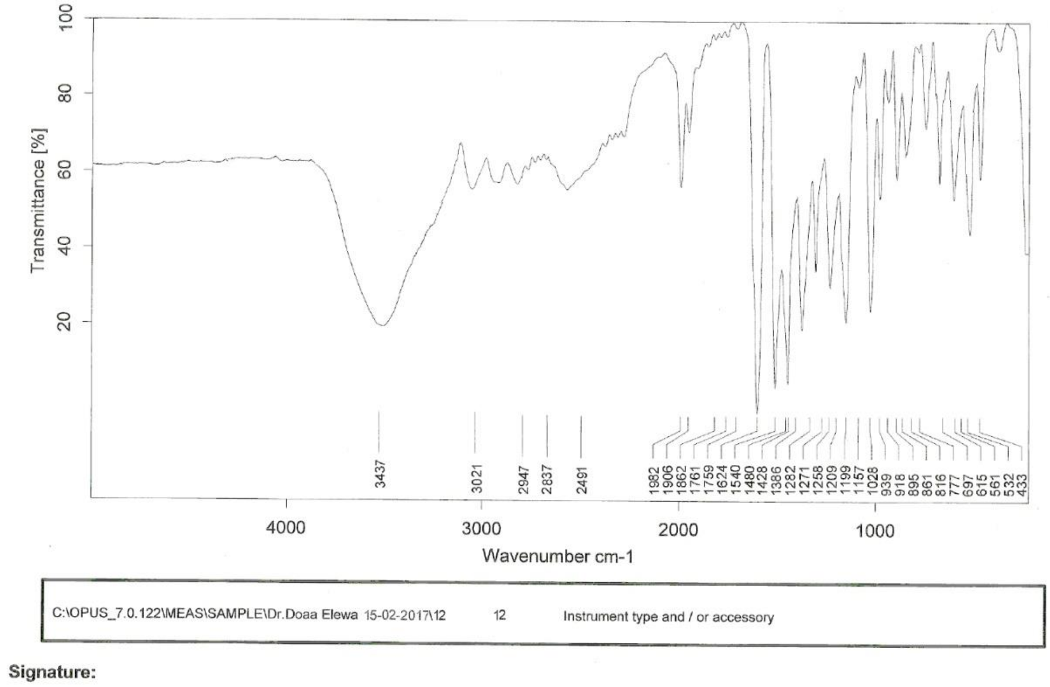

S45: IR spectrum of **7.**

Docking studies of metabolites on the active site of PTP1B (1G7F)

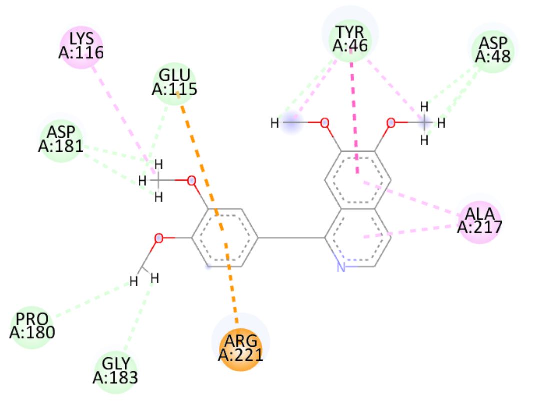

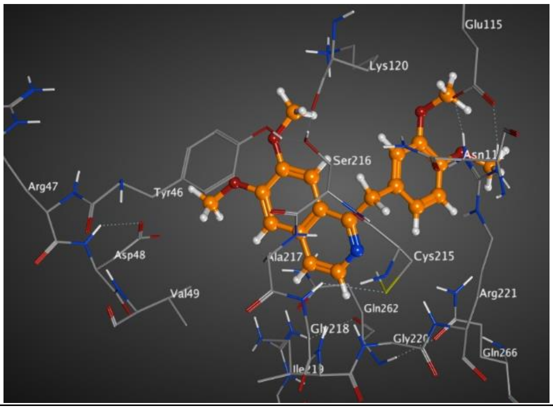

S46. 2D (left) and 3D (right) patterns demonstrate the binding interaction of metabolite **papaverine** into the active site of PTP1B (PDB ID: 1G7F).

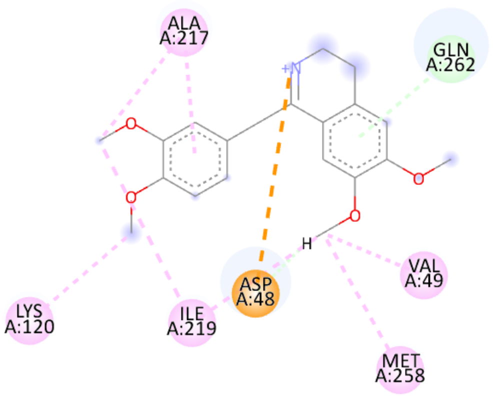

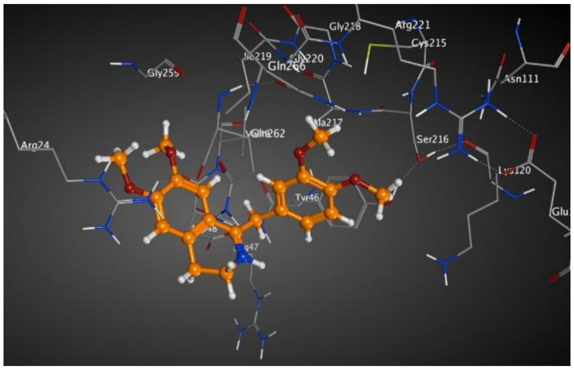

S47. 2D (left) and 3D (right) patterns demonstrate the binding interaction of metabolite **1** into the active site of PTP1B (PDB ID: 1G7F).

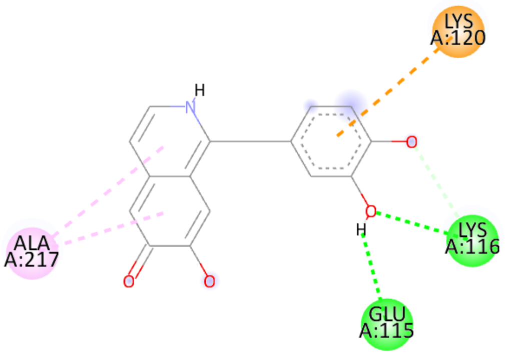

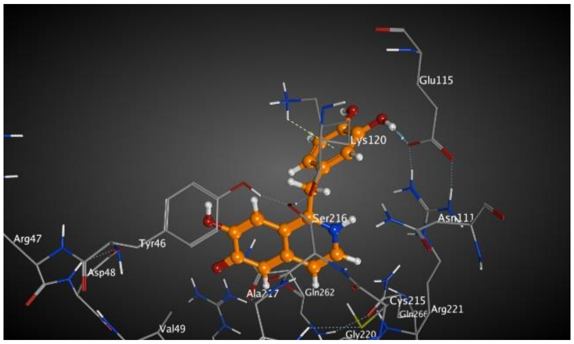

S48. 2D (left) and 3D (right) patterns demonstrate the binding interaction of metabolite **2** into the active site of PTP1B (PDB ID: 1G7F).

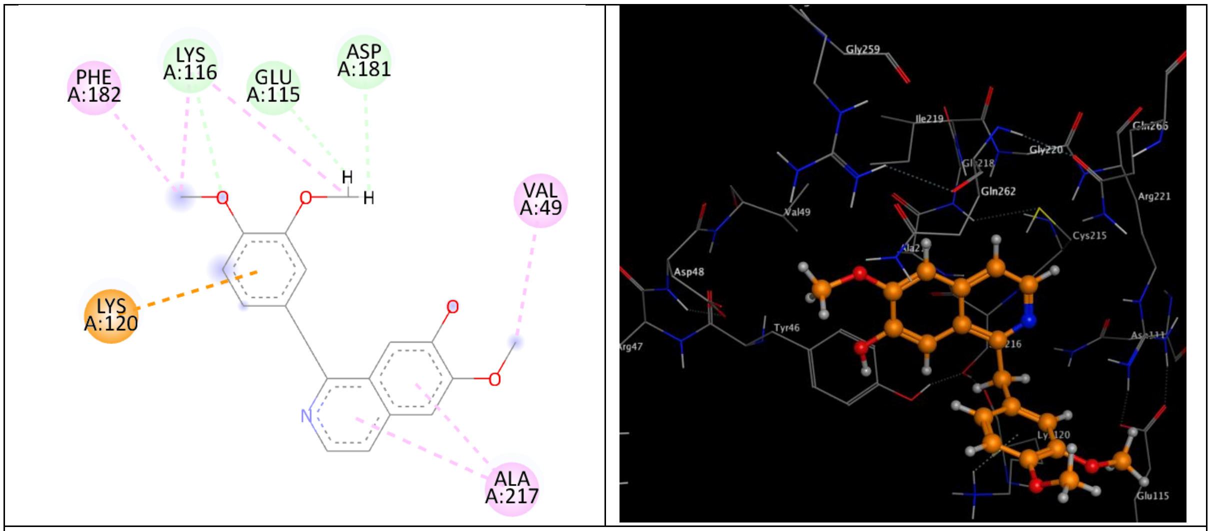

S49. 2D (left) and 3D (right) patterns demonstrate the binding interaction of metabolite **3** into the active site of PTP1B (PDB ID: 1G7F).

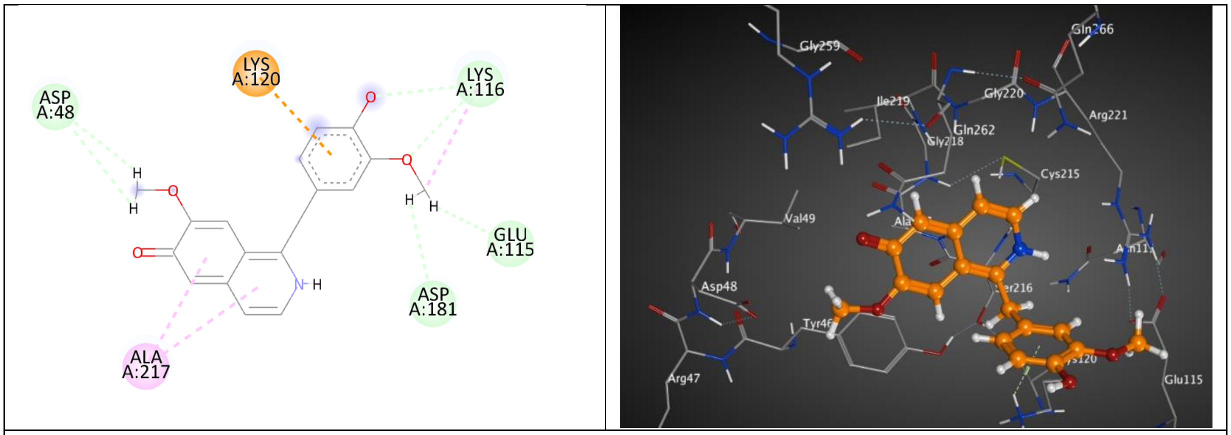

S50. 2D (left) and 3D (right) patterns demonstrate the binding interaction of metabolite **4** into the active site of PTP1B (PDB ID: 1G7F).

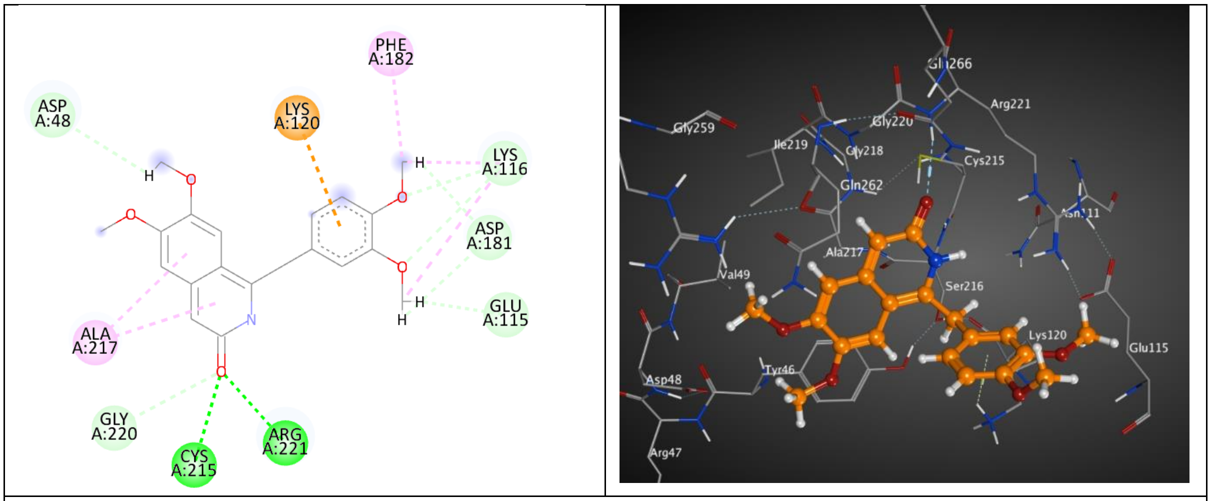

S51. 2D (left) and 3D (right) patterns demonstrate the binding interaction of metabolite **5** into the active site of PTP1B (PDB ID: 1G7F).

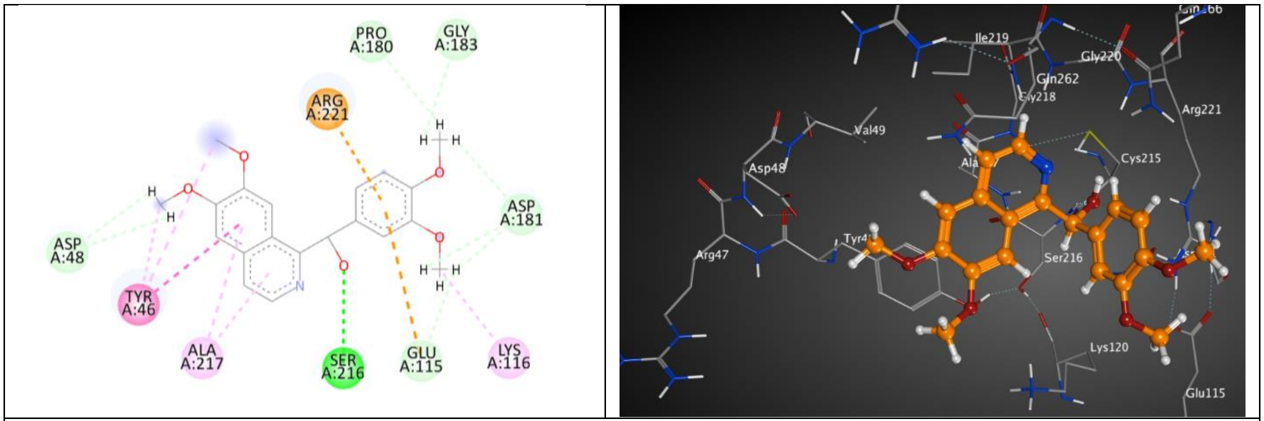

S52. 2D (left) and 3D (right) patterns demonstrate the binding interaction of metabolite **6** into the active site of PTP1B (PDB ID: 1G7F).

Docking studies of metabolites on the active site of α-glucosidase (3A4A)

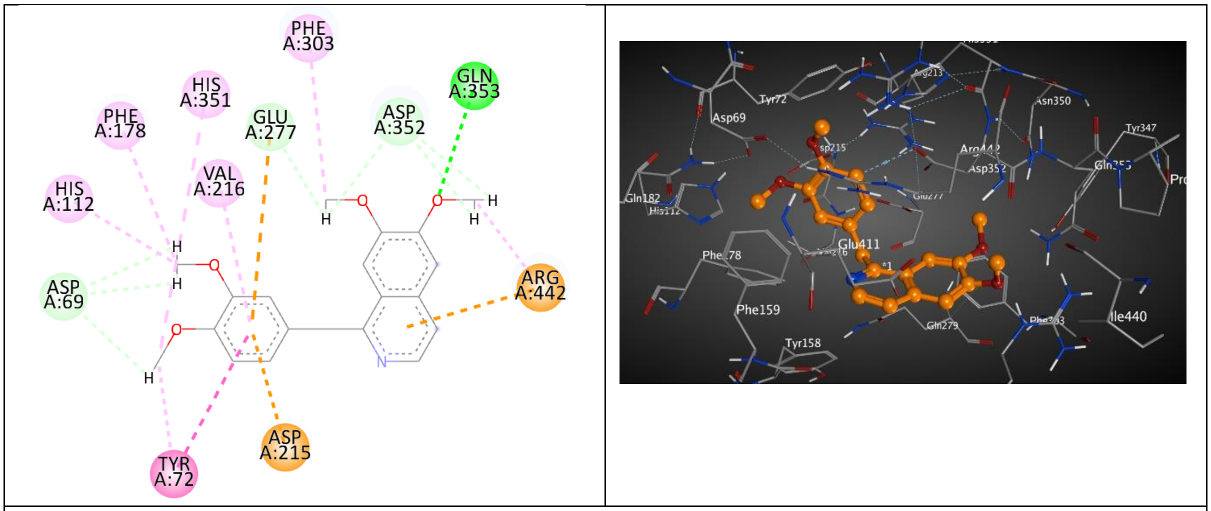

S53. 2D (left) and 3D (right) patterns demonstrate the binding interaction of metabolite **papaverine** into the active site of α-glucosidase (PDB ID: 3A4A).

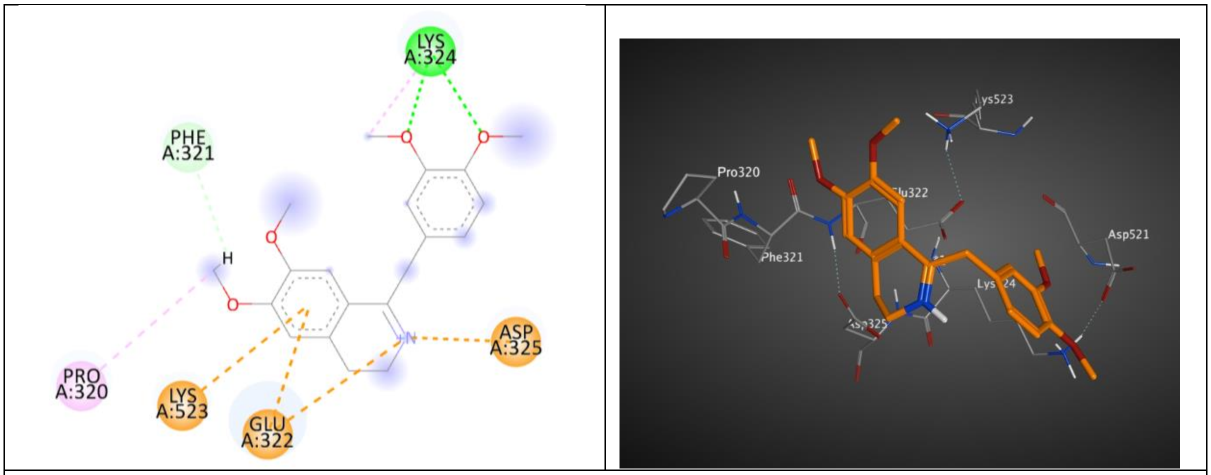

S54. 2D (left) and 3D (right) patterns demonstrate the binding interaction of metabolite **1** into the active site of α-glucosidase (PDB ID: 3A4A).

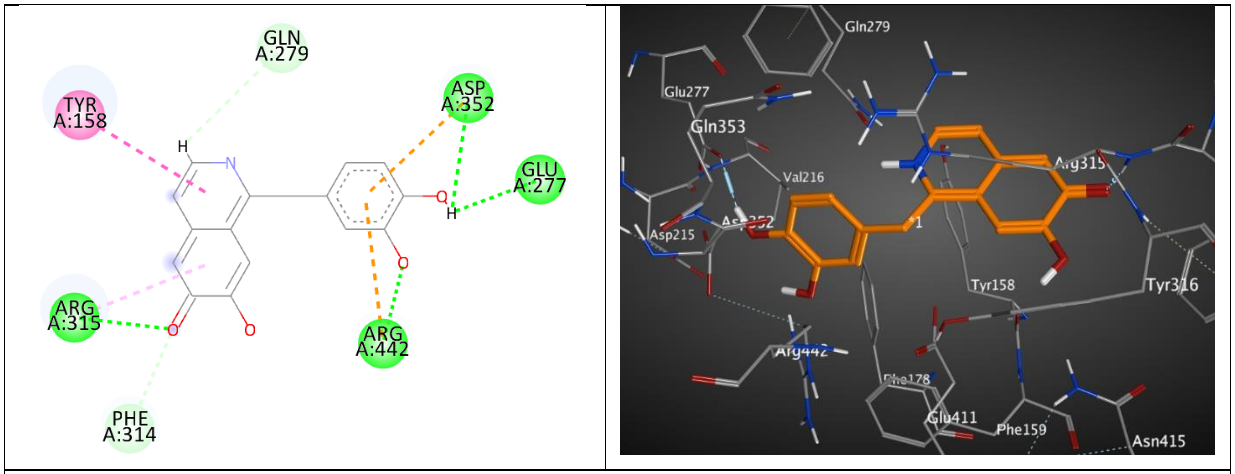

S55. 2D (left) and 3D (right) patterns demonstrate the binding interaction of metabolite **2** into the active site of α-glucosidase (PDB ID: 3A4A).

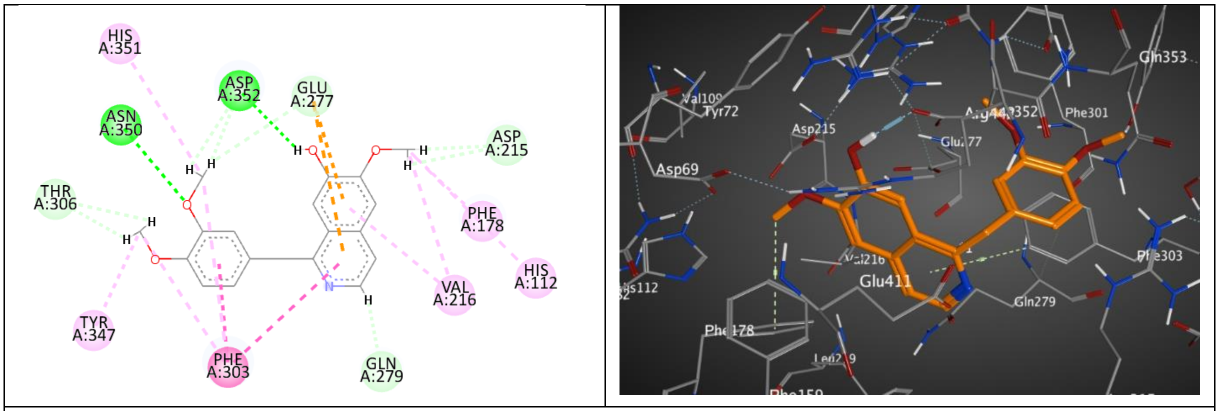

S56. 2D (left) and 3D (right) patterns demonstrate the binding interaction of metabolite **3** into the active site of α-glucosidase (PDB ID: 3A4A).

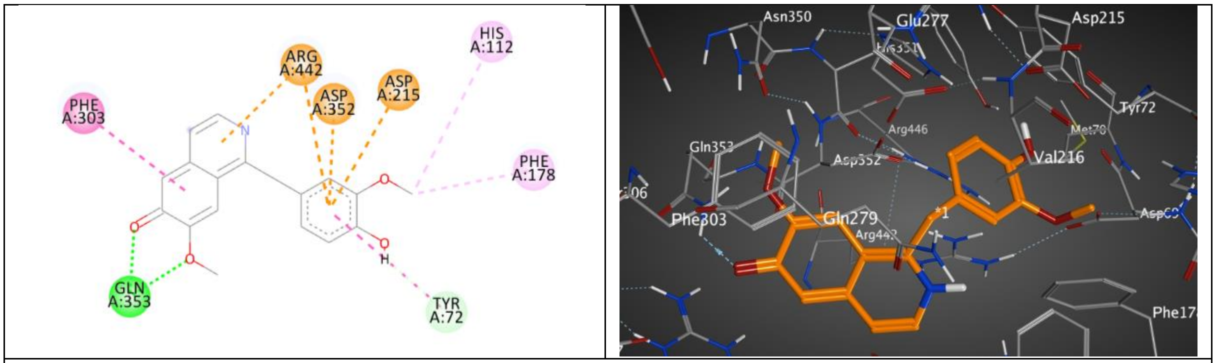

S57. 2D (left) and 3D (right) patterns demonstrate the binding interaction of metabolite **4** into the active site of α-glucosidase (PDB ID: 3A4A).

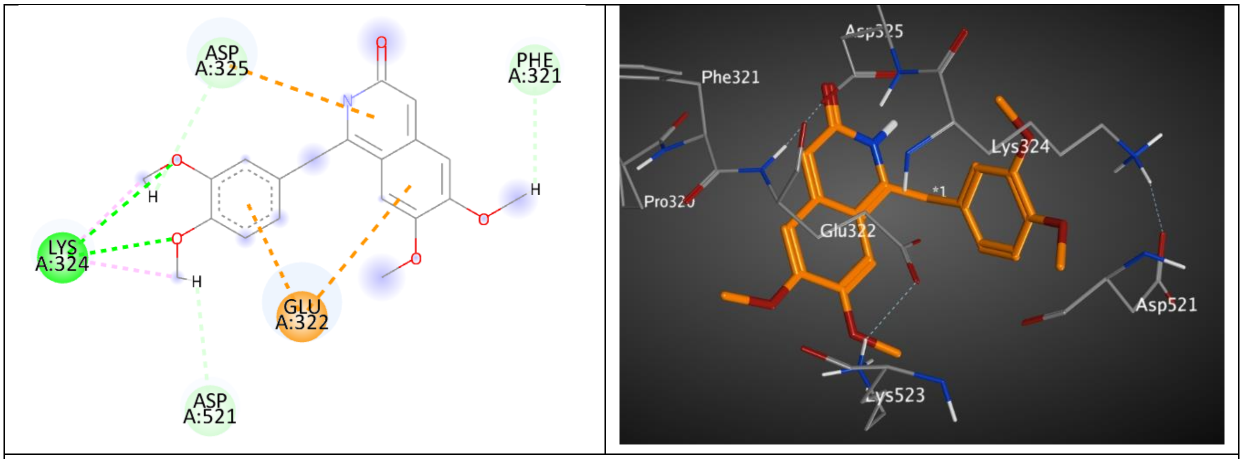

S58. 2D (left) and 3D (right) patterns demonstrate the binding interaction of metabolite **5** into the active site of α-glucosidase (PDB ID: 3A4A).

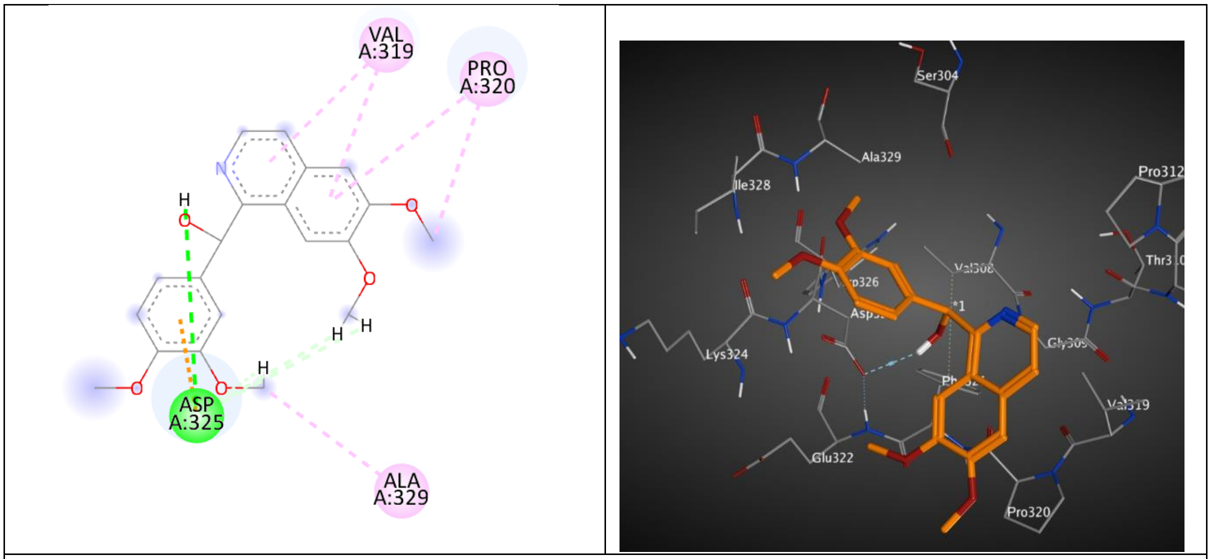

S59. 2D (left) and 3D (right) patterns demonstrate the binding interaction of metabolite **6** into the active site of α-glucosidase (PDB ID: 3A4A).

Docking studies of metabolites on the active site of lipase (PDB: 1LPB)

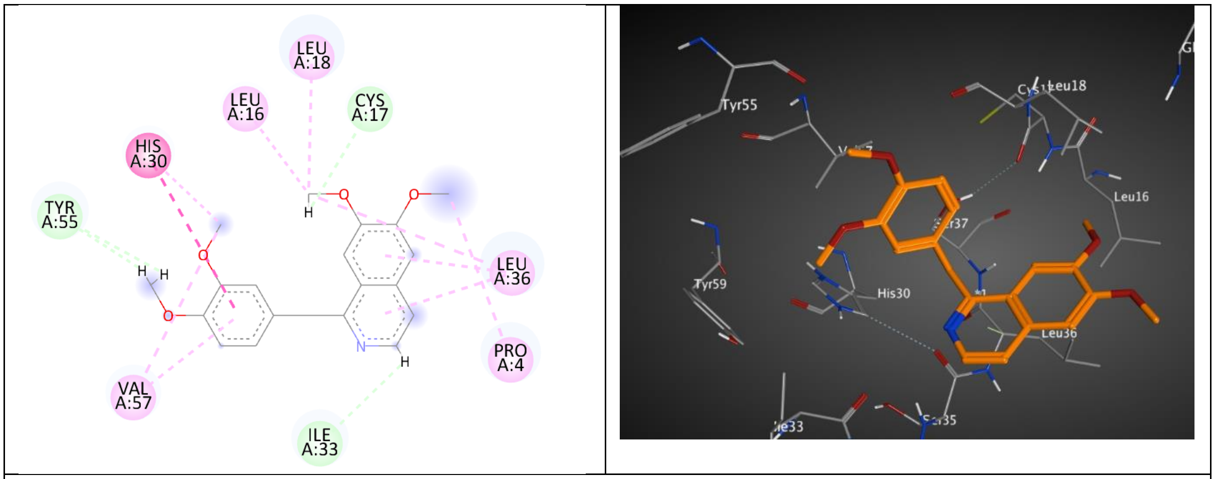

S60. 2D (left) and 3D (right) patterns demonstrate the binding interaction of metabolite **papaverine** into the active site of lipase (PDB ID: 1LPB).

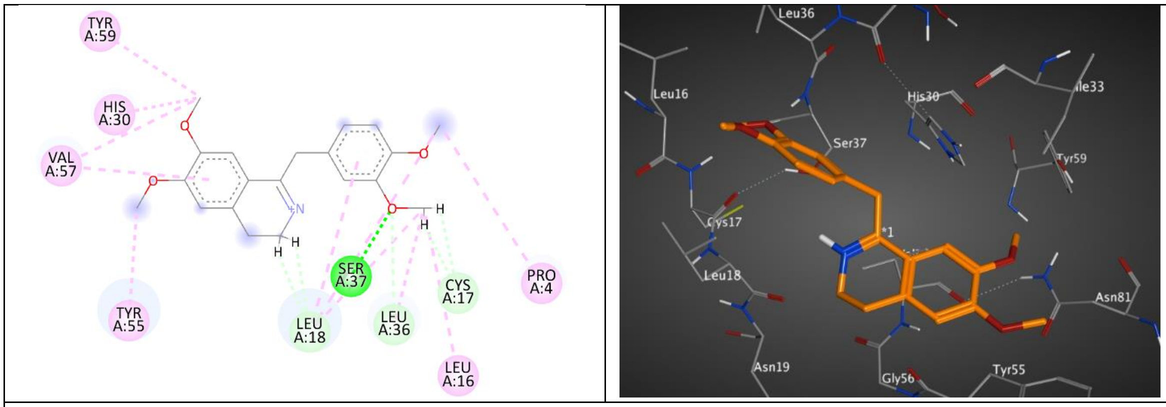

S61. 2D (left) and 3D (right) patterns demonstrate the binding interaction of metabolite **1** into the active site of lipase (PDB ID: 1LPB).

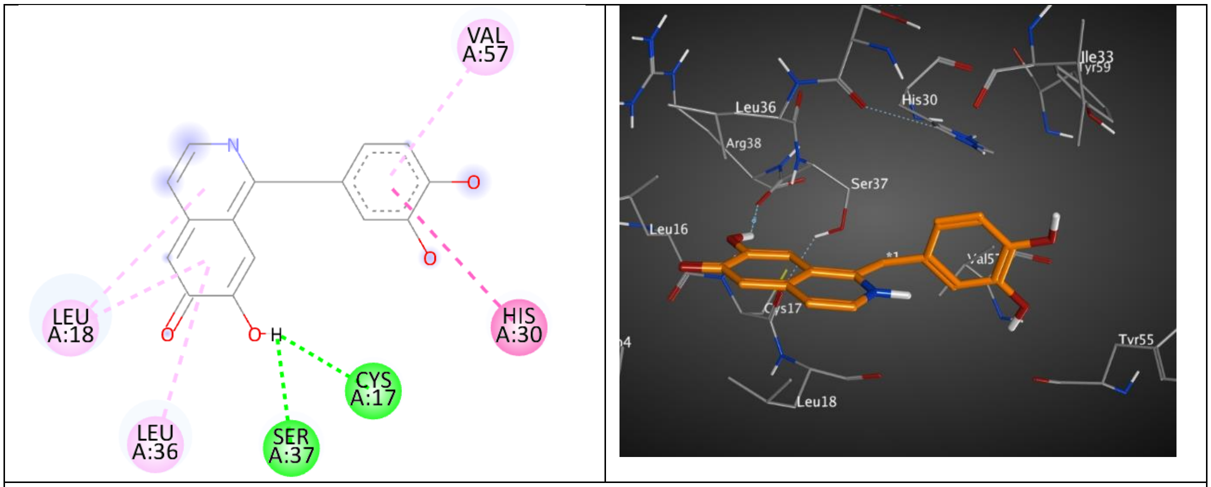

S62. 2D (left) and 3D (right) patterns demonstrate the binding interaction of metabolite **2** into the active site of lipase (PDB ID: 1LPB).

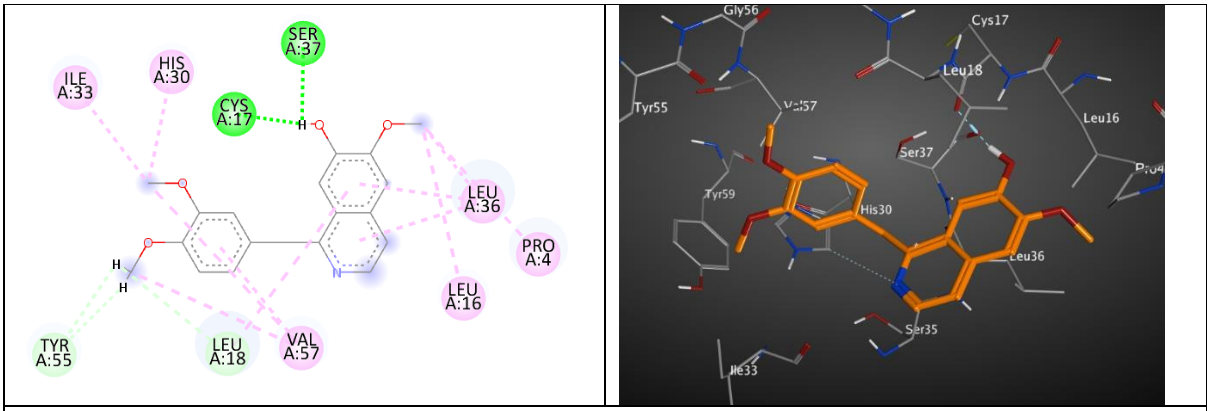

S63. 2D (left) and 3D (right) patterns demonstrate the binding interaction of metabolite **3** into the active site of lipase (PDB ID: 1LPB).

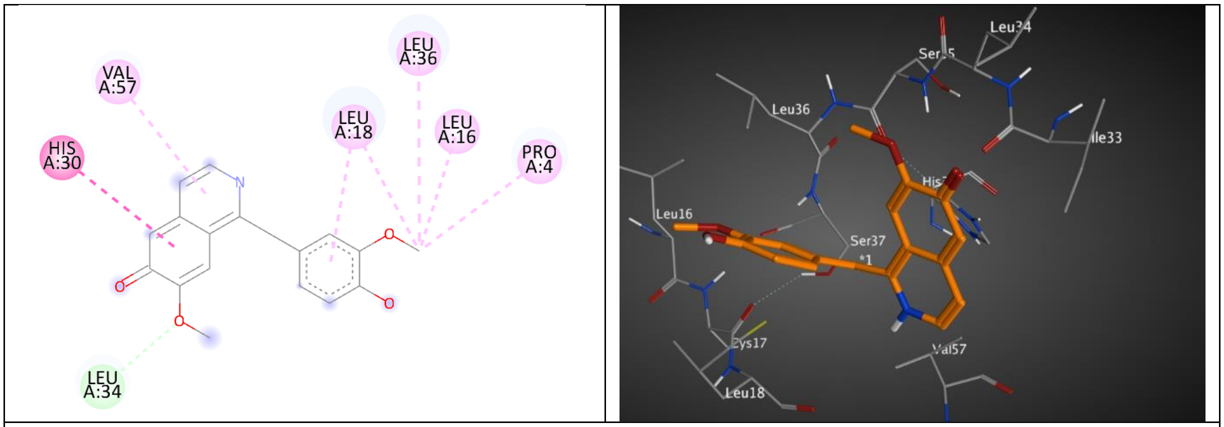

S64. 2D (left) and 3D (right) patterns demonstrate the binding interaction of metabolite **4** into the active site of lipase (PDB ID: 1LPB).

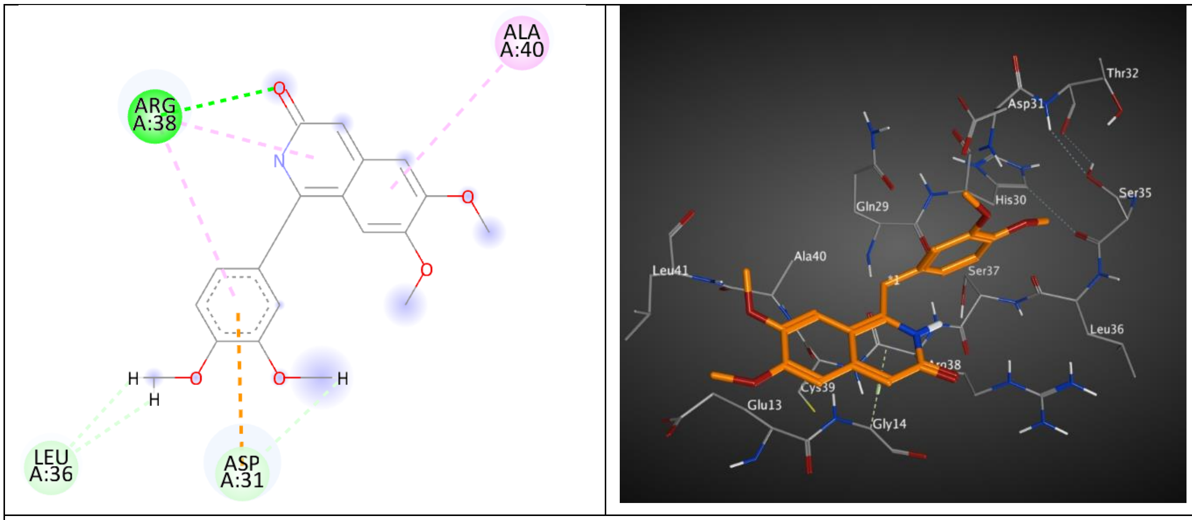

S65. 2D (left) and 3D (right) patterns demonstrate the binding interaction of metabolite **5** into the active site of lipase (PDB ID: 1LPB).

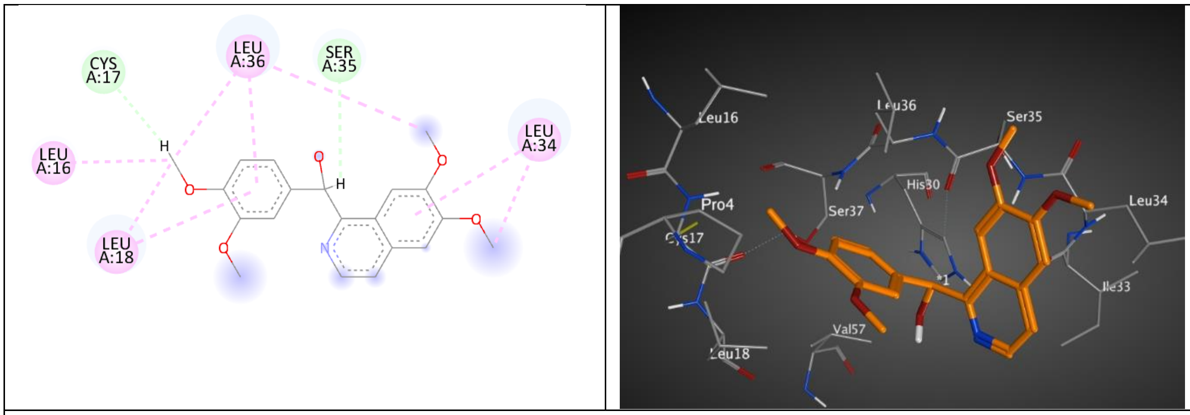

S66. 2D (left) and 3D (right) patterns demonstrate the binding interaction of metabolite **6** into the active site of lipase (PDB ID: 1LPB).
